# Surface Density-Dependent Interactions between Photoactivated Sensory Rhodopsin 2 and Its Transducer

**DOI:** 10.1021/acsomega.5c12030

**Published:** 2026-02-02

**Authors:** Tatsuya Sakamoto, Jingyi Tang, Soichiro Kato, Insyeerah Binti Muhammad Jauhari, Tatsuro Nishikino, Yuji Furutani

**Affiliations:** † Department of Life Science and Applied Chemistry, 12982 Nagoya Institute of Technology, Showa-ku, Nagoya 466-8555, Japan; ‡ OptoBioTechnology Research Center, 12982 Nagoya Institute of Technology, Showa-ku, Nagoya 466-8555, Japan

## Abstract

Sensory rhodopsin 2 from *Natronomonas pharaonis* (*p*SRII) is a heptahelical transmembrane protein that functions as a photosensor of a microbe with its cognate transducer protein, *p*HtrII, which is a bihelical transmembrane protein with a long cytoplasmic domain for regulation of flagella rotation. It was revealed that *p*SRII and *p*HtrII form a 2:2 complex, and the photoreaction induces tilting of the sixth helix (the F helix) in *p*SRII, which rotates a transmembrane helix of *p*HtrII. The conformational change is presumably transferred to the cytoplasmic domain through HAMP domains, which are ubiquitous for chemosensory systems. X-ray crystallography on the *p*SRII complex with *p*HtrII truncated C-terminal domain did not resolve a HAMP domain because of its structural flexibility. Thus, the involvement of the HAMP domain in the protein–protein interactions between the *p*SRII and *p*HtrII complexes is still elusive. Here, we applied surface-enhanced infrared spectroscopy to *p*SRII and *p*SRII fused with *p*HtrII­(1-159) including a HAMP domain or *p*HtrII­(1-83) excluding the domain to study structural changes under a physiological membrane orientation. Interestingly, the light-induced difference spectrum of *p*SRII-*p*HtrII­(1-159) was strongly attenuated in the amide I region in a surface-density-dependent manner. The suppression was observed almost similarly in *p*SRII-*p*HtrII­(1-83), suggesting that the conformation change of *p*SRII-*p*HtrII in the membrane region would be a primary key factor for the initial signal transduction from *p*SRII to *p*HtrII without interaction with the HAMP domain.

## Graphical abstract

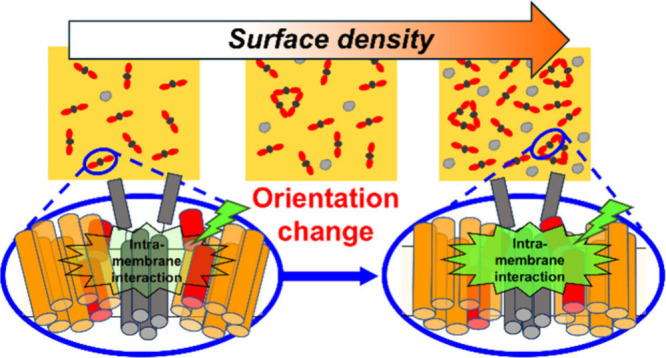

## 1 Introduction

Light detection in the biological system starts with a photoresponsive protein, such as rhodopsin, in our vision, which possesses a retinal chromophore for capturing light.^1,2^ Photoisomerization of the chromophore (e.g., 11-*cis* to all-*trans* in vision) induces a conformational change of the receptor protein and transfers the signal to another protein through protein–protein interaction (PPI). Rhodopsin in vision binds to a trimeric G protein (transducin) as a transducer. In archaea, microbial rhodopsins with all-*trans* retinal chromophore propagate light signal through a cognate halobacterial transducer protein, HtrI or HtrII.^3–5^ For the negative phototaxis response, sensory rhodopsin 2 (SRII) functions as a light sensor and interacts with HtrII, which regulates the kinases (CheA, CheB, CheR, CheW, and CheY) involved in the two-component system^6^ (Figure 1a). HtrII interacts with CheW and regulates the phosphorylation of CheA, which is also regulated by CheB and CheR. Finally, phosphorylated CheY interacts with a flagellar motor to change the swimming direction of an archaeal cell.^7^

**Figure 1:**
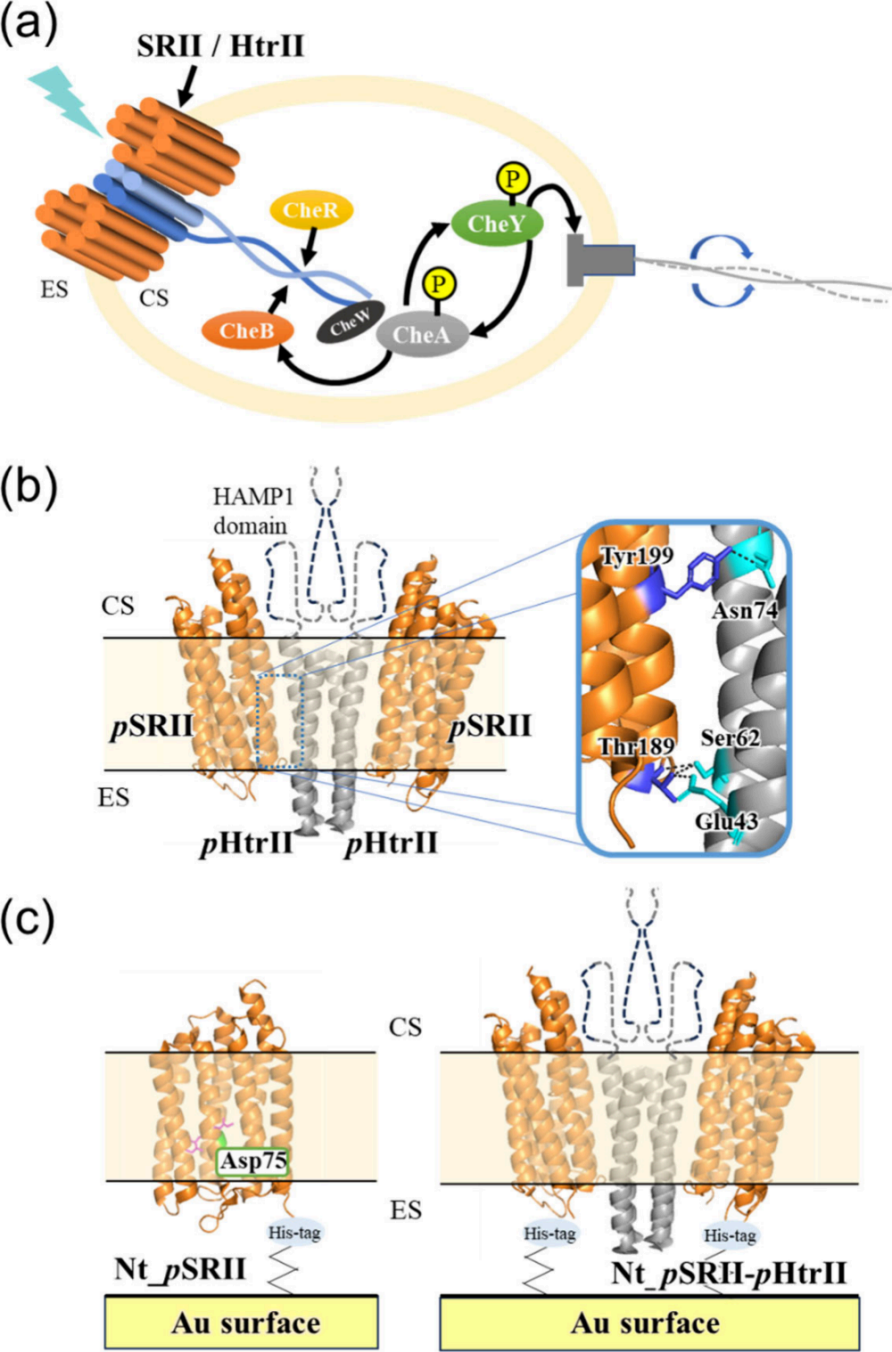
(a) Schematic illustration of a bacterial cell possessing a light sensor, the SRII/HtrII complex (2:2), and kinases (CheA, CheB, CheR, CheW, and CheY) involved in the signal transduction, a two-component system, for regulating rotation of a flagellar motor. (b) X-ray crystal structure of *p*SRII complexed with truncated *p*HtrII. The HAMP1 domain was not solved by X-ray crystallography, which was included in our expression plasmid. *p*SRII interacts with *p*HtrII through hydrogen bonds shown in the inset. (c) The orientation of the *p*SRII and *p*SRII-*p*HtrII fusion proteins used in this study. His-tag at the N-terminus binds the Ni-NTA linker on the gold surface. The ribbon models of *p*SRII and *p*HtrII from the PDB codes of 3QAP (*p*SRII monomer) and 1H2S (*p*SRII/*p*HtrII complex) are colored orange and gray, respectively. CS: cytoplasmic side, ES: extracellular side.

The all-*trans* to 13-*cis* isomerization of the retinal chromophore initiates the photocyclic reaction of SRII, which sequentially forms intermediates K, L, M, and O and returns to the original state.^8–24^ The protonated retinal Schiff base (PRSB), which forms a covalent bond with a lysine residue on the seventh helix (G helix), transfers the proton to its counterion, an aspartate residue on the third helix (C helix) upon formation of the M intermediate,^15^ which is the active state of SRII and changes the interaction with HtrII.^24–33^ The precise molecular events were mostly studied in SRII found from *Natronomonas pharaonis* (*p*SRII), which can be heterologously expressed in *E. coli* and purified under low-salt conditions. For their feasibility for biophysical experiments, the molecular mechanisms of the photoreaction of *p*SRII and its signal transduction to *p*HtrII have been extensively studied.^9–55^

Previous X-ray crystallographic studies solved the atomic structures of *p*SRII alone^34,35,45^ and its complex with truncated *p*HtrII.^36,40,48,53^ *p*SRII/ *p*HtrII forms a 2:2 complex (dimer of dimers) in the unphotolyzed state and the tilt of the F helix in *p*SRII rotates the second transmembrane helix (TM2) in *p*HtrII upon formation of the M intermediate state,^40^ which was initially observed by electron paramagnetic resonance (EPR) spectroscopy.^9,13,21^ According to several reports, the light signal is transmitted to the C-terminal of *p*HtrII, where the histidine kinase CheA coupled with CheW is attached, through two HAMP domains.^33,41,46,47,49–52,55–57^ The HAMP1 domain was included in the expression construct for X-ray crystallography; however, the atomic details were not clarified^36,40,48,53^ (Figure 1b). It has been widely accepted that a HAMP domain is composed of two α helical regions that interact side by side and are connected to a linker region.^37–39,41,46,47,49–52,55–57^ Therefore, the ambiguity in the X-ray structure was probably due to its structural flexibility. More precise information on PPI was monitored by light-induced difference Fourier transform infrared (FTIR) spectroscopy.^16,17,19,26–28^ The initial proton transfer from the PRSB to its counterion, Asp75, induces the conformation change of *p*SRII (formation of the intermediate M)^15^ and the hydrogen bond between Tyr199 in *p*SRII and Asn74 in *p*HtrII was strengthened^26,27^ (Figure 1b). Furthermore, the tilting of the F helix of *p*SRII is attenuated by the interaction with *p*HtrII at room temperature upon the formation of the M intermediate.^26,28^

Surface-enhanced infrared spectroscopy (SEIRAS) is a powerful tool for measuring infrared absorption spectra of a monolayer of molecules formed on the surface of a gold thin film.^58,59^ The enhancement is generally limited to ∼10 nm from the surface and highly dependent on the orientation of a vector of a vibrational transition moment of a molecule.^59^ That is, the vibrational mode with the vibrational transition moment perpendicular to the gold surface is highly enhanced. For the transmembrane helical protein, the axis of α helix tends to parallel the membrane normal.^60,61^ When transmembrane proteins are attached to the surface of the thin gold film with orientation perpendicular to the surface plane, the amide I mode of the helix (∼1654 cm^–1^) is highly enhanced, but the amide II mode (∼1550 cm^–1^) is not.^60,61^ The amide I mode is mainly composed of the C=O stretching modes of the peptide backbone, while the amide II mode is mainly composed of the N–H bending modes.^62,63^ For the transmembrane helical protein oriented perpendicularly to the gold surface, the C=O bonds vibrate along the helix axis; hence, the vibrational transition moments also orient along the normal of the gold surface. On the other hand, the N–H groups laterally vibrate with vibrational transition moments perpendicular to the helix axis and the normal of the gold surface.^60,61^

In this study, SEIRAS was applied with light-induced difference spectroscopy to analyze the interactions of *p*SRII and *p*HtrII upon the formation of the active state (M intermediate). In particular, the samples were attached to the gold surface by a Ni-NTA linker at the N-terminus of *p*SRII, allowing the molecules to orient the extracellular side close to the surface and the intracellular side toward the opposite (Figure 1c). Therefore, PPIs are expected to occur in a similar manner on a native biological membrane. Furthermore, the *p*HtrII domain truncated at the 83rd or 159th position was fused to the C-terminus of *p*SRII (Figure S1). The former construct (*p*SRII-*p*HtrII­(1-83)) does not contain the HAMP1 domain, but the latter construct (*p*SRII-*p*HtrII­(1-159)) does. The effect of the HAMP1 domain on PPIs was investigated. It should be noted that SEIRAS enhancement is expected to extend throughout the membrane-embedded region, although it may be slightly less sensitive to the HAMP1 domain. Infrared absorption of *p*SRII increased the intensities dependent on surface density as expected, while those of *p*SRII-*p*HtrII­(1-83) and *p*SRII-*p*HtrII­(1-159) changed not only the intensities but also the spectral shapes. The light-induced difference spectra of *p*SRII-*p*HtrII­(1-83) and *p*SRII-*p*HtrII­(1-159) were attenuated under the high surface density, which were basically similar to the previous experimental results obtained from randomly oriented samples of *p*SRII/ *p*HtrII^28^ (*p*SRII and the truncated *p*HtrII­(1-159) were prepared separately) and the fusion protein *p*SRII-*p*HtrII­(1-120).^26^ On the basis of these experimental results, the molecular mechanisms of signal transduction between the *p*SRII and *p*HtrII domains are discussed, especially in the integral and peripheral regions of the membrane.

## 2 Materials and Methods

### 2.1 Construction of the *p*SRII-*p*HtrII Fusion Proteins

To construct the plasmid, pET21a_*Nt*_*pSRII*, cording a hexa-histidine tag for affinity purification fused in the N-terminal of *p*SRII (*Nt_pSRII*), the fragment of the *pSRII* gene was amplified from the plasmid pET21a_*Ct*_*pSRII* by PCR using a forward primer containing the NdeI site and hexa-histidine codons (AAGGA­GATATA­CATAT­GCACC­ACCAC­CACCA­CCACGT­GGGAC­TTACG­ACCCTC) and a reverse primer containing SacI and *Eco*RI sites (GACGG­AGCTCG­AATTCT­TAGTCG­GCGACC­GCAG). The pET21a vector was digested with NdeI and *Eco*RI, and then, the restricted vector fragment was purified by electrophoresis. The *Nt_pSRII* gene fragment was inserted into the amplified pET21a vector fragment using In-fusion cloning (Takara), and named pET21a_*Nt_pSRII*.

To construct the plasmid pET21a_*pSRII-pHtrII_1-159*, the vector pET21a with the *his*_6_-*pSRII* gene was amplified from the plasmid pET21a_*Nt_pSRII* by PCR using a forward primer containing *Eco*RI and SacI sites (TAAGA­ATTCGA­GCTCC­GTCGAC) and a reverse primer containing the polypeptide linker “ASASNGASA” as used in previous studies^16,26^ (GACGCG­CCTGCG­GTCGCCG­ACGCGAG­CGCGAG­CAACGG­CGCG­AGCGC­GATGTC­GCTGAA­CGTA). Furthermore, the 1-159 truncated fragment of the *p*HtrII gene (*pHtrII_1-159*) was amplified from plasmid pET21a_*pHtrII* by PCR using a forward primer (ATGTC­GCTGAA­CGTATC­ACGG) and a reverse primer containing *Eco*RI and SacI sites (GAGGAG­ATCAACA­CGGAAC­TATAAG­AATTCG­AGCTC). The *pHtrII_1-159* fragment was inserted into the amplified pET21a vector fragment with the *his*_6_ *-pSRII* gene using In-fusion cloning and named pET21a_*pSRII-pHtrII_1-159*.

Construction of the plasmid pET21a_*pSRII-pHtrII*_*1-83*, coding a 1-83 truncation of *p*HtrII fused in the C-terminal of His_6_-*p*SRII, is performed using the QuikChange method (Agilent) from the plasmid pET21a_*pSRII-pHtrII_1-159* using a forward primer (CTTGTC­GACGG­AGCTCG­AATTCT­TAGCCC­AGCGTG­GCAGCA­AC) and a reverse primer (GTTGC­TGCCAC­GCTGGG­CTAAGAA­TTCGAG­CTCCGTC­GACAAG).

All constructs were confirmed by DNA sequencing. The amino acid sequences are shown in Figure S1. The transformation of *E. coli* was performed by using a standard method using CaCl_2_.

### 2.2 Preparation of the *p*SRII Protein

*p*SRII possessing a hexa-histidine tag at the N-terminus was expressed in the *E. coli* C41­(DE3) strain. Bacterial cells were grown overnight in 10 mL of 2x YT medium (1.6% (w/v) Tryptone, 1.0% (w/v) Yeast extract, and 0.5% (w/v) NaCl) in shaker flasks at 37 °C and 180 rpm, inoculated in 1.0 L of 2x YT medium, and cultured at 37 °C adding air. When the density of the cells at OD_660_ approximately reached 0.3, isopropyl β-D-thiogalactopyranoside (IPTG) and all-*trans* retinal were added to final concentrations of 1 mM and 10 μM, respectively, to induce protein overexpression and cultured for 4 h at 37 °C. Cells were harvested by centrifugation and were washed in buffer A (50 mM Tris­(hydroxymethyl)­aminomethane (Tris)-HCl [pH 8.0], 300 mM NaCl and 5 mM imidazole). To break the cells, the suspension was treated twice with a French press (No. 5501-M, Ohtake Works) at 100 MPa. After unbroken cells were removed by low-speed centrifugation, the samples were ultracentrifuged at 118,000 × *g* for 1 h. The pellet was suspended and homogenized in buffer B (50 mM 2-morpholinoethanesulfonic acid (MES)-NaOH [pH 6.5], 300 mM NaCl, 5 mM imidazole) and stored at −80 °C until use. The frozen sample was thawed and stirred in a water bath. To solubilize the suspension, n-dodecyl-β-d-maltoside (DDM) was added to the final concentration of 1% (w/v) and stirred overnight at 4 °C. The insoluble materials were removed by centrifugation (118,000 × *g* for 30 min). The resulting supernatants were mixed with 10 mL of nickel-nitrilotriacetic acid (Ni-NTA) agarose resin (QIAGEN) in a polypropylene column by a batch method. After the supernatant was eluted in the column, buffer C (50 mM MES-NaOH [pH 6.5], 300 mM NaCl, 50 mM imidazole, 0.1% (w/v) DDM) was added to wash the column. To elute the His-tag protein from the resin, buffer E (50 mM Tris-HCl [pH 7.0], 300 mM NaCl, 150 mM imidazole, and 0.1% (w/v) DDM) was added, and the mixture was collected.

### 2.3 Preparation of the *p*SRII-*p*HtrII Fusion Proteins

*E. coli* C41 (DE3) cells carried the plasmid based on pET21a coded *pSRII-pHtrII_1-159* or *pSRII-pHtrII_1-83* with a hexa-histidine tag at its N-terminus cultured for protein expression. The cultivation, solubilization, and purification with a Ni-NTA column were modified from the protocol described in the previous section. For further purification, size-exclusion chromatography was applied after Ni-NTA column purification.

The bacterial cells were grown overnight in 20 mL of LB medium (1.0% (w/v) Tryptone, 0.5% (w/v) Yeast extract, and 0.5% (w/v) NaCl) at 37 °C, inoculated in 2.0 L of 2x YT medium, and cultured at 37 °C. When the cell density at OD_660_ approximately reached at 0.4, IPTG and all-*trans* retinal were added to a final concentration of 1.0 mM and 10 μM, respectively, to induce protein overexpression and cultured for 1 day at 37 °C. Cells were harvested by centrifugation, the cells weight was measured, and suspended in 7 mL of buffer F (50 mM Tris-HCl [pH 8.0], 300 mM NaCl and 5 mM Imidazole) per 1 g (wet weight) of cells. To break the cells, the suspension was treated twice by the French press at 100 MPa. After unbroken cells were removed by low-speed centrifugation, the samples were ultracentrifuged at 118,000 × *g* for 1 h. The pellet was suspended in the original volume of buffer F and stored at −80 °C until use. The frozen sample was thawed and stirred in the water bath. To solubilize the suspension, DDM was added to the final concentration of 1% (w/v) and stirred for at least 60 min at 4 °C. Insoluble materials were removed by centrifugation (118,000 × *g* for 30 min). The resulting supernatants were mixed with 30 mL of Ni-NTA agarose resin (Fujifilm Wako) in a polypropylene column by batch method. After eluting the supernatant in the column, 150 mL of buffer B′ (50 mM Tris-HCl [pH 8], 300 mM NaCl, 10 mM Imidazole, 0.1% (w/v) DDM) was added to wash the column. To elute the His-tag protein from the resin, 40 mL of buffer C’ (50 mM Tris-HCl, pH 7.0, 300 mM NaCl, 300 mM imidazole, and 0.1% (w/v) DDM) was added and collected in 5 mL fractions. The His-tag affinity-purified *p*SRII-*p*HtrII­(1-159) or *p*SRII-*p*HtrII­(1-83) proteins were concentrated in 0.4 mL using an Amicon device with 30 kDa cutoff (Merck Millipore), loaded onto a size-exclusion column (Superdex 200 increase 10/300 column, Citiva) equilibrated with buffer D (100 mM NaH_2_PO_4_ [pH7.0], 150 mM NaCl and 0.1% (w/v) DDM) and eluted with buffer D at the flow rate of 0.5 mL min^–1^. The peak fractions were collected and measured by 500 and 280 nm absorption using ultraviolet–visible (UV–vis) spectrometry (V-730, Jasco).

### 2.4 Vacuum Deposition of a Thin Gold Film for SEIRAS

A vacuum chamber was used for the deposition of a thin gold film on a Si crystal (ATR crystal used for VeeMaxII, PIKE Technology, USA), which was performed similarly to previous papers.^64,65^ To avoid oil mist contamination, in this study, a turbomolecular pump (HiPace 80, PFEIFFER, Germany) was used connected to a dry vacuum pump (NeoDry15 × 10^–1^, Kashiyama, Japan). The thickness of the gold thin film was monitored by a QCM sensor (008–101-G10, 6 MHz gold-coated crystal) connected to a deposition monitor (XTM/2 deposition monitor, INFICON). A 9.0 nm gold thin film was evaporated onto the Si crystal at a deposition rate of 0.1 nm/min up to 1.0 and 0.2 nm/min beyond 1.0 nm, in a vacuum of 0.50 × 10^–5^ Torr or less. The Si crystal and QCM were cooled by circulating water at 25 °C (Si crystal) and 24 °C (QCM) from thermostatic baths (ALPHA RA8, LAUDA).

### 2.5 Modification of the Thin Gold Surface for Protein Adsorption with Ni-NTA Linker

A gold thin film used for SEIRAS was modified by Ni-NTA according to the previous report.^61,65^ Each modification step was monitored *in situ* by SEIRAS. First, the gold thin film was exposed to a 200 μL solution of 2 mg/mL dithiobis (succinimidyl propionate) (DTSP, Thermo Fisher Scientific) in dimethyl sulfoxide (DMSO) for 20 min. After washing the surface with 200 μL of DMSO four times, the DMSO solution was dried in a nitrogen stream. Next, the TSP monolayer was immersed in 200 μL aqueous solution of 40 mg/mL of N-(5-amino-1-carboxypentyl)-iminodiacetic acid (AB-NTA, Dojindo) in 0.5 M K_2_CO_3_ buffer (pH 9.8). The excess AB-NTA was removed by washing the surface with 200 μL of high-purity water three times and then once with a 200 μL solution of 50 mM acetate buffer (pH 5.5). The NTA-modified surface was incubated with a 200 μL solution of 40 mM NiSO_4_ in 50 mM acetate buffer (pH 5.5). In this process, a Ni^2+^ ion is coordinated via three carboxylates and a tertiary amine in an NTA linker. After washing the surface with 200 μL of buffer D twice, 200 μL of the protein-solubilized solution was added and left for 1 h 40 min to connect the hexa-histidine-tag *p*SRII or *p*SRII-*p*HtrII fusion proteins with Ni-NTA linkers. The sample concentration for the adsorption process was 0.25, 2.5, or 25 μM (0.01, 0.1, or 1.0 absorbance at λ_max_ (∼500 nm) of each sample). After the protein monolayer was formed on the gold surface, excess protein-solubilized solution was removed and the surface was washed with 200 μL buffer D three times. Reconstitution of the protein into the lipid bilayer was carried out by adding 200 μL solution of 2 mg/mL L-α-phosphatidylcholine (egg-PC, SIGMA) in PC buffer (50 mM HEPES, 140 mM NaCl, 0.2 w/v% DDM (pH 7.0)) and then removing the DDM detergent with 20 mg of adsorbent (SM-2, Biobeads, Bio-Rad). Finally, the protein monolayer embedded in the lipid bilayer was washed by dialysis buffer without DDM until all excess lipid was removed.

### 2.6 Light-Induced Difference Spectroscopy with SEIRAS

Infrared spectra of SEIRAS were measured with a FTIR spectrometer (Vertex70, Bruker) at a resolution of 2 cm^–1^, sample scan: 32 scans, background scan: 128 scans, save data range: 4000–700 cm^–1^, aperture: 4 mm, acquisition range: 6000–0 cm^–1^, and averaged 100 times for each condition. The protein-modified Si ATR crystal was placed on an ATR accessory (VeeMaxII, PIEK Technology, USA). The incident angle was set to 48 degrees. An IR polarizer was not installed to increase the signal-to-noise ratio. The sample chamber temperature was kept at 20 °C by placing a copper jacket with circulating water from a thermostat bath (Alpha RA8, LAUDA). The sample was irradiated with 30 mW light from a metal halide light source (IMH-250, SIGMAKOKI, Japan) through a 500 nm interference filter, and the dark and light procedure cycle (30 s in the dark and 10 s of light irradiation) was repeated 100 times to calculate the light-dark difference spectrum. For accumulating the light state (M intermediate), measurements of the light spectra were performed during light irradiation. Then, the optical filter was changed to 560 nm; measurements were made in the same way as for 500 nm; and the difference spectrum was calculated. Finally, the light-dark difference spectrum at 560 nm was subtracted from the light-dark difference spectrum at 500 nm to remove the spectral change caused by photoreactions other than protein samples (photoresponse of the Si surface covered by a thin gold film).

## 3 Results

### 3.1 Infrared Absorption Spectra of *p*SRII and *p*SRII-*p*HtrII­(1-159) in the Amide I and II Regions Recorded by SEIRAS at Different Protein Concentrations

SEIRAS makes it possible to measure a protein monolayer on a metal surface (usually Au).^43,44,60,61,64–68^ The constructs of *p*SRII and *p*SRII-*p*HtrII contain a hexa-histidine tag at the N-terminus (Figure S1). Therefore, the extracellular sides of the *p*SRII and *p*SRII-*p*HtrII molecules are expected to attach to the gold surface through a Ni-NTA linker, as shown in Figure 1c. Protein orientations are also expected to be uniformly aligned on the surface.^60,61^ The situation would be different from the proteins reconstituted in liposomes with a random orientation. In the physiological membrane in bacteria, the N-terminus of *p*SRII protrudes outward from the membrane, and the C-terminus of *p*HtrII is prolonged to the intracellular side. Therefore, SEIRAS is an ideal IR measurement method for analyzing the molecular interactions between *p*SRII and *p*HtrII with physiological orientations in the lipid bilayer.

The peptide backbone is composed of the C=O and N–H groups in the peptide bonds, which exhibit strong infrared absorption bands called amide I (1695–1623 cm^–1^) and amide II (∼1550 cm^–1^).^62,63^ SEIRAS monitored the adsorption processes of *p*SRII and *p*SRII-*p*HtrII­(1-159) in the amide I and II regions (Figures S2 and S3). The final infrared absorption spectra recorded after incubation for 100 min are shown in Figure 2. As expected, the intensities of the amide I (1654 cm^–1^) band of *p*SRII increased depending on the concentrations of *p*SRII (Figure 2c). The spectral shapes were unchanged (Figure 2a), suggesting that the conformation of *p*SRII did not change with increasing surface density on the Ni-NTA-modified gold surface. The results were demonstrated more clearly by calculating the second derivative of the spectra (Figures 2e,g).

**Figure 2:**
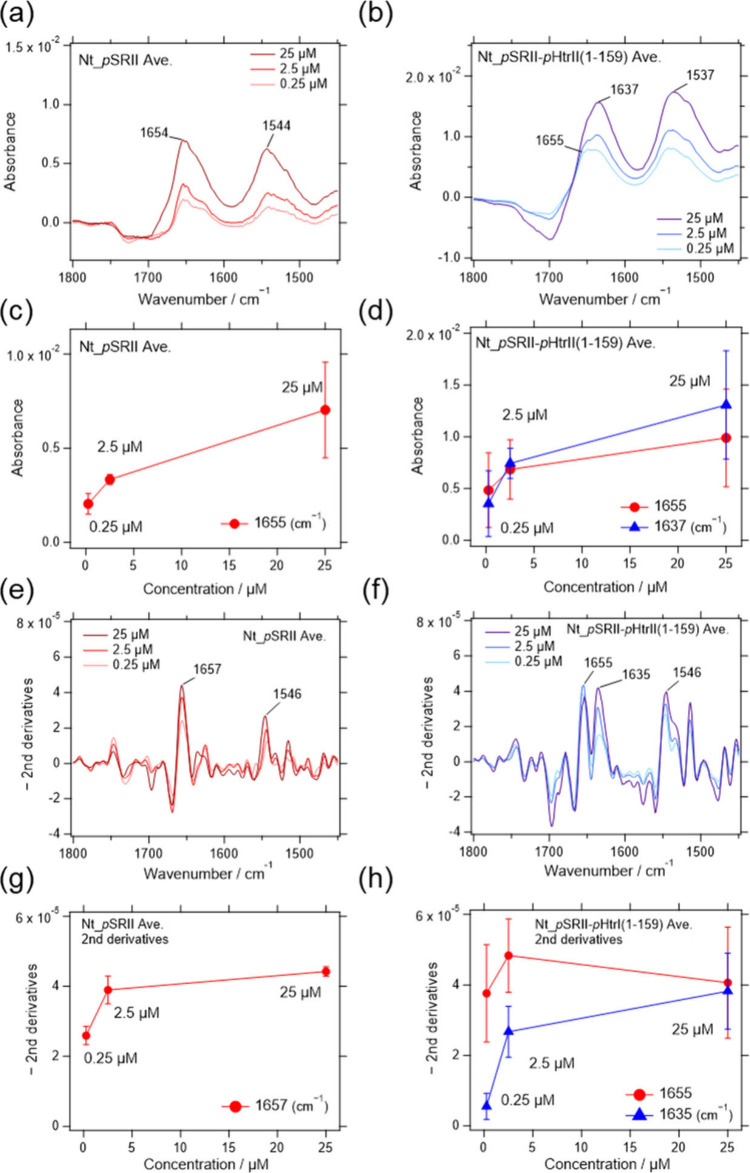
Infrared absorption spectra of (a) *p*SRII and (b) *p*SRII-*p*HtrII­(1-159) obtained by SEIRAS Sample concentrations for adsorption on the Ni-NTA-modified gold surface were 0.25, 2.5, and 25 μM. Three and four experiments were averaged for *p*SRII and *p*SRII-*p*HtrII­(1-159), respectively. (c, d) Absorption at the peaks (*p*SRII; 1654 cm^–1^, *p*SRII-*p*HtrII­(1-159); 1637 and 1655 cm^–1^) are plotted against the sample concentrations. (e, f) The second derivatives multiplied by −1 were calculated for each sample. (g, h) The peak values in the second derivative spectra were plotted as in (c, d).

However, *p*SRII-*p*HtrII­(1-159) exhibited unexpected spectral changes dependent on the protein concentrations. As shown in Figure 2b, the amide I bands showed a broad shape compared with *p*SRII. Furthermore, the peak positions gradually changed from 1655 to 1637 cm^–1^ (Figure 2b,d). The results were also confirmed in the second derivative spectra (Figure 2f,h). The peak position of an amide I band is generally used for analysis of the secondary structure of protein.^62,63^ A protein with helical structures exhibits a strong peak at ∼1654 cm^–1^, while that with β-sheet structures exhibits a strong peak at 1634 cm^–1^.^62,63^ Therefore, it might suggest that *p*SRII-*p*HtrII­(1-159) increases the β-sheet structure with an increase in the surface density. However, it is not likely that, according to the X-ray structure of the 2:2 complex of *p*SRII and *p*HtrII^36^ (Figure 1b), the major parts are composed of helical structures except for a small β-sheet structure in the extracellular loop in *p*SRII. Furthermore, such spectral changes were not observed in the absorption spectra of *p*SRII-*p*HtrII­(1-159) reconstituted in liposomes (Figure S4). Therefore, concentration-dependent spectral changes of *p*SRII-*p*HtrII­(1-159) were only observable by SEIRAS. The optical purities of *p*SRII and *p*SRII-*p*HtrII fusion proteins are shown in Figure S5. The absorbance ratio at 278 and 498 nm is 1.56 for *p*SRII and 2.23 for *p*SRII-*p*HtrII­(1-159), indicating that *p*SRII-*p*HtrII­(1-159) has slightly lower purity than *p*SRII. No obvious contamination by unidentified proteins was observed in conventional FTIR spectroscopy (Figure S4); however, an unidentified protein component was detected in SEIRAS measurements. This may suggest that the contaminating proteins were preferentially adsorbed onto the Ni-NTA-modified gold surface.

A noticeable negative band around 1700 cm^–1^ is observed, particularly in *p*SRII-*p*HtrII fusion proteins. This feature may arise from the removal of interfacial water molecules—specifically their O–H bending vibrations—upon protein adsorption to the surface, as well as from changes in the refractive index associated with an increase in the amount of material on the gold surface. The depletion of water molecules is also supported by the presence of a negative band in the 3000–3600 cm^–1^ region (Figure S6).

As shown in Figure S7, no disappearance of the amide I band was observed after reconstitution. The band at 1631 cm^–1^ is attributed to the O–H bending vibrations of water that were excluded during the formation of the lipid bilayer. The band at 1743 cm^–1^ likely originates from the C=O stretching vibration of the ester groups in the lipid molecules. In the C–H stretching region, bands at 2924 and 2853 cm^–1^ are clearly assigned to the CH stretching vibrations of the alkyl chains of the lipids.

These spectra were recorded after re-establishing the background following the immobilization of *p*SRII or the *p*SRII–*p*HtrII fusion proteins on the Ni-NTA-modified surface. If protein denaturation or desorption had occurred during the lipid reconstitution process, we would expect detectable changes in the amide I or II regions. However, no such changes were observed, further supporting that the proteins remained structurally intact throughout the reconstitution procedure.

### 3.2 Comparison of Light-Induced Structural Changes of *p*SRII and *p*SRII-*p*HtrII­(1-159) with Different Surface Concentrations Monitored by SEIRAS

The active state of *p*SRII, M intermediate, is formed by irradiation with blue light.^15,28^ To investigate the molecular mechanism of signal transmission from *p*SRII to *p*HtrII, blue light (500 nm) was irradiated on *p*SRII and *p*SRII-*p*HtrII­(1-159) modified on the gold surface and reconstituted into a lipid bilayer. Light-induced difference spectra were measured by SEIRAS. However, the light-induced difference spectrum recorded between the continuously illuminated condition and the dark state containing spectral changes of components adsorbed on the Si surface—such as the Ni-NTA linker, proteins, lipids, and interfacial water molecules, is influenced by the photoresponse of the silicon substrate, as shown in Figure S8a. Therefore, the light-induced difference spectra with green light illumination (560 nm), which does not activate *p*SRII, were measured as a reference. The double difference spectra between these two illumination conditions were calculated as the final light-induced difference spectra of *p*SRII and *p*SRII-*p*HtrII­(1-159). For example, the light-induced difference spectrum of *p*SRII was obtained as shown in Figure S8b. Upon the formation of the M intermediate, the protonated retinal Schiff base is deprotonated, and its counterion, Asp75, receives the proton.^15^ The positive band at 1762 cm^–1^ was previously assigned to the carboxyl C=O stretching band of Asp75.^15^ The negative band at 1543 cm^–1^ was assigned to the ethylenic C=C stretching mode of the retinal chromophore in the dark state of *p*SRII. These features are very characteristic of the light-induced difference spectrum between the M intermediate and the dark state of *p*SRII.

The protein concentration dependencies on the light-induced difference spectra of *p*SRII and *p*SRII-*p*HtrII­(1-159) were analyzed (Figure 3). The amplitude of the spectral change of *p*SRII increased with increasing protein concentration (Figure 3a,c), which is reasonable because the surface density of *p*SRII increases, as seen in the absorption spectra (Figure 2a). That is, the amplitudes of the amide I bands between 1642 and 1663 cm^–1^ (Figure 3c) showed a trend similar to those of the amide I bands at 1655 cm^–1^ in the absorption spectra (Figure 2c). However, the amplitude of the spectral change of *p*SRII-*p*HtrII­(1-159) decreased despite the increase in protein concentration (Figure 3b,d). Consequently, the amplitudes of the amide I bands between 1640 and 1662 cm^–1^ (Figure 3d) showed a trend opposite those of the amide I bands at 1637 and 1655 cm^–1^ in the absorption spectra (Figure 2d). Our results suggest that the fractional coverage of *p*SRII-*p*HtrII­(1-159) on the Ni-NTA-modified gold surface decreases with increasing the protein concentration. Increased adsorption of β-sheet-rich contaminants (monitored at 1637 cm^–1^) likely dilutes the surface density of *p*SRII-*p*HtrII­(1-159), resulting in a smaller light-induced difference signal. Could PPIs between *p*SRII and *p*HtrII further influence the surface packing and signal amplitude?

**Figure 3:**
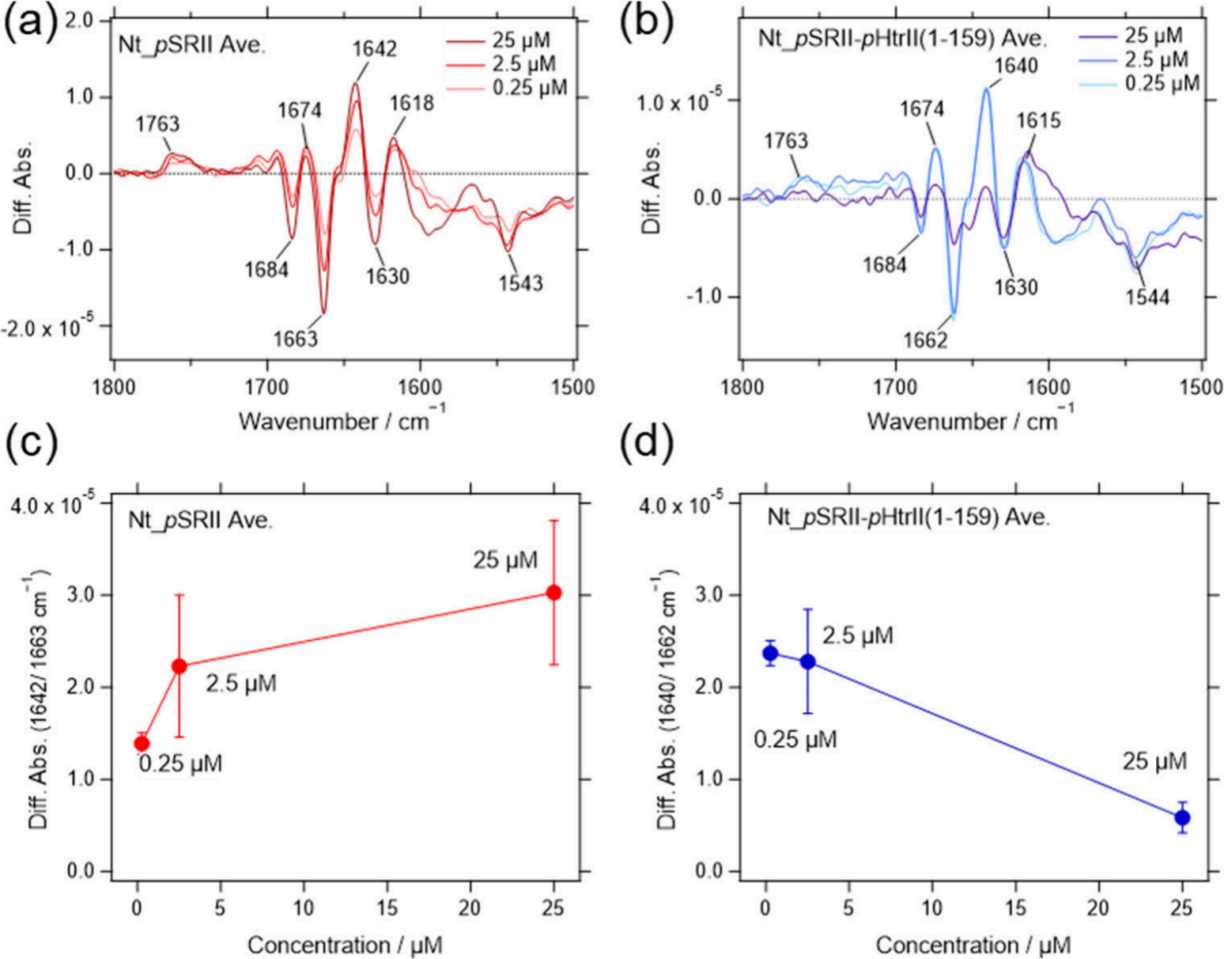
Light-induced difference infrared spectra of (a) *p*SRII and (b) *p*SRII-*p*HtrII­(1-159) obtained by SEIRA spectroscopy. The spectra were recorded in the samples adsorbed on the Ni-NTA-modified gold surface at protein concentrations of 0.25, 2.5, and 25 μM. The difference in absorbance between 1642 and 1663 cm^–1^ is plotted with a function of protein concentration for (c) *p*SRII and (d) *p*SRII-*p*HtrII­(1-159) (d). The error bars are the standard deviations calculated from three independent experiments (*p*SRII) and four (*p*SRII-*p*HtrII (1-159)).

The concentration-dependent effects of the formation of the complex with the *p*HtrII­(1-159) domain were analyzed by comparing the light-induced difference spectra of *p*SRII and *p*SRII-*p*HtrII­(1-159) after normalization using the negative peak at 1543 cm^–1^ (Figure 4). Interestingly, the attenuation of the peaks at 1641 and 1662 cm^–1^ was clearly dependent on the protein concentration applied in the adsorption process, as shown in Figure 4a, c, e. That is, the decrease in the light-induced difference spectra in *p*SRII-*p*HtrII­(1-159) at a high protein concentration cannot be solely explained by the existence of contaminated proteins. It was also confirmed in the double difference spectra in Figure 4b,d,f. The pair of maximums at 1648 and 1665 cm^–1^ was evidently observed in Figure 4b (25 μM). The small pair of peaks at ∼1683 and ∼1690 cm^–1^ was probably originated from the C=O stretching vibration of Asn74 at *p*HtrII, which was assigned in the previous studies.^26,27^ The C=O stretching vibration of Asn74 was generally observed at concentrations of 0.25, 2.5, and 25 μM. These results suggest that the hydrogen-bonding interaction of Asn74 in *p*HtrII with Tyr199 in *p*SRII changes similarly independent of the surface density of the complex. However, the PPI that prevents the tilting motion of the F helix in *p*SRII is highly dependent on the surface density. Attenuation of the spectral change in the amide I region was also confirmed in the reconstituted liposomes of *p*SRII-*p*HtrII­(1-159) (Figure S9) as reported for the complex of *p*SRII and *p*HtrII­(1-159) proteins prepared separately^28^ and the fusion protein *p*SRII-*p*HtrII­(1-120).^26^ In liposomes, the complexes of *p*SRII-*p*HtrII­(1-159) formed structures of higher order (trimer of dimers) observed by electron microscopy.^54^ The formation of higher-order structures may be hampered in *p*SRII-*p*HtrII­(1-159) dispersedly adsorbed on the Ni-NTA modified gold surface.

**Figure 4:**
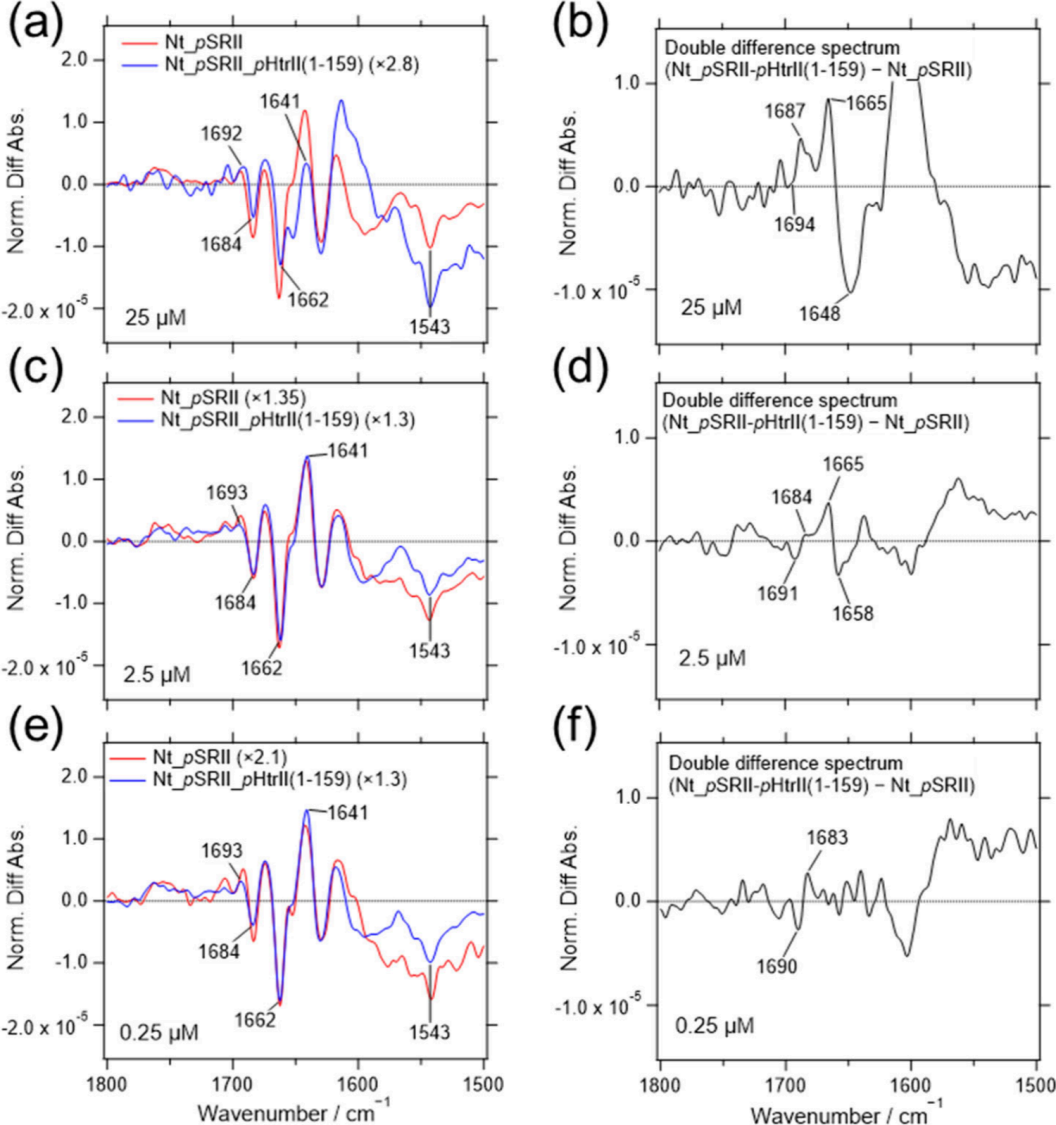
Comparison of the light-induced difference spectra of *p*SRII and *p*SRII-*p*HtrII­(1-159) after normalization The light-induced difference spectra (a, c, e in the left panel) were normalized based on the substantial amplitude of the negative peak at the retinal ethylenic C=C stretching vibration at 1543 cm^–1^. The normalization factors are shown in parentheses after the sample names. The double difference spectra (b, d, and f in the right panel) were calculated by subtracting the difference spectrum of *p*SRII from those of the *p*SRII-*p*HtrII­(1-159). Sample concentrations for adsorption were 25, 2.5, and 0.25 μM as indicated in each panel.

### 3.3 The HAMP1 Domain Is Not Critical for Protein–Protein Interactions between *p*SRII and *p*HtrII in the Membrane-Embedded Domains

The light-induced difference spectra with different surface densities elucidated the PPIs between *p*SRII and *p*HtrII truncated at the 159th position. Concentration-dependent suppression of light-induced conformation change after the formation of the M intermediate is reflected in the decrease in the positive band at 1641 cm^–1^ and the negative band at 1662 cm^–1^ in accordance with the increase in the protein concentration (Figure 4). In previous FTIR spectroscopy on the *p*SRII/*p*HtrII­(1-159) complex reconstituted in liposomes, the attenuation of the spectral change in the amide I bands was observed in a temperature-dependent manner.^28^ This difference highlights the characteristic features of the SEIRAS experiments. It should be mentioned that attenuation was not observed in the *p*SRII complex with the G83C or G83F mutant of *p*HtrII­(1-159).^28^ Therefore, the previous report stressed the importance of the HAMP1 domain, which plays a crucial role in the suppression of the tilting movement of the F helix of *p*SRII upon M formation.^28^

Interestingly, a concentration-dependent increase in the amide I band was observed similarly at 1637 cm^–1^ in *p*SRII-*p*HtrII­(1-83) lacking the HAMP1 domain (Figure 5a–d). This result excluded the possibility that the HAMP1 domain formed β-sheet structure in the condensed environment, which is consistent with previous experimental and theoretical studies reporting that the HAMP domains are mainly constructed from α helical structures.^33,41,47,49–52,55,57^ It also supports the assumption that the increase in the 1637 cm^–1^ band is probably due to contaminated proteins.

**Figure 5:**
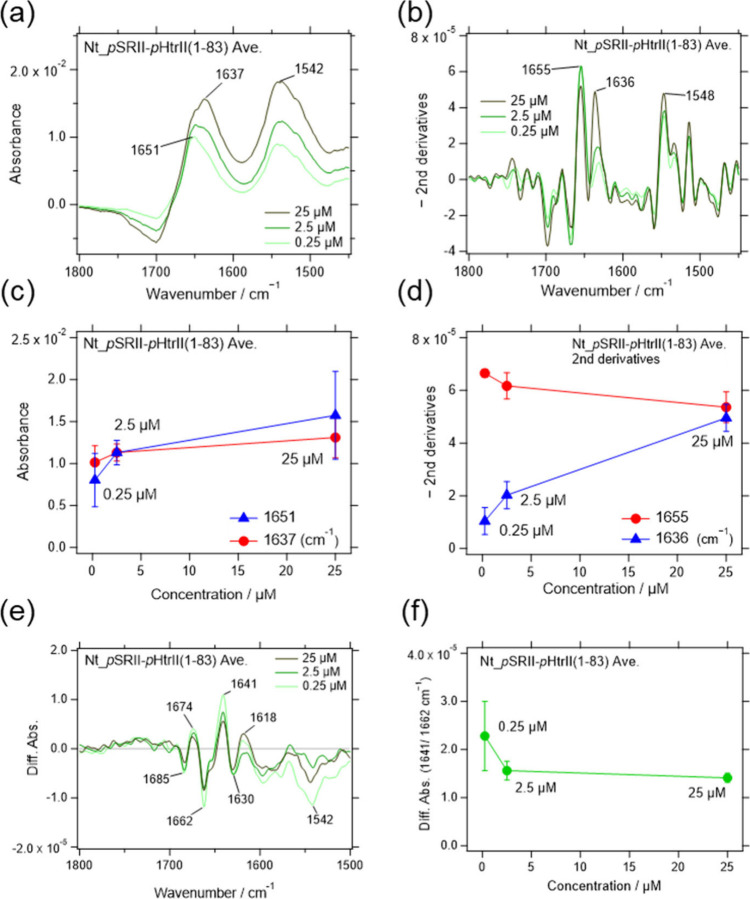
Infrared absorption spectra of *p*SRII-*p*HtrII­(1-83) obtained by SEIRAS. (a) Sample concentrations for adsorption on the Ni-NTA-modified gold surface were 0.25, 2.5, and 25 μM. Three experiments were averaged. (b) The second derivatives multiplied by −1 were calculated for each sample. (c) Absorption at the peaks (*p*SRII-*p*HtrII­(1-83); 1637 and 1651 cm^–1^) are plotted against the sample concentrations. (d) The peak values in the second derivative spectra were plotted similarly to (c). (e) The light-induced difference infrared spectra of *p*SRII-*p*HtrII­(1-83) were obtained by SEIRA spectroscopy. (f) The amplitudes between the positive peak at 1641 cm^–1^ and the negative peak at 1662 cm^–1^ are plotted as a function of the sample concentrations.

The amide I bands at 1641 and 1662 cm^–1^ in the light-induced difference spectra of *p*SRII-*p*HtrII­(1-83) decreased their intensities with increased protein concentration (Figure 5e,f). The reduction was slightly reduced compared to that observed in *p*SRII-*p*HtrII­(1-159) (Figure 3b,d), suggesting that the interaction with the HAMP1 domain partially contributes to the suppression of the tilt of the F helix in *p*SRII. This fact was confirmed in this SEIRAS experiment for the first time. The fusion protein *p*SRII-*p*HtrII­(1-83) would force the *p*HtrII transmembrane domain to lie adjacent to the *p*SRII domain, which cannot be achieved by the complex formation of separately prepared *p*SRII and *p*HtrII­(1-83) due to their weak affinity.^18^

After normalizing the light-induced difference spectra and calculating the double difference spectra between the *p*SRII and *p*SRII-*p*HtrII­(1-83) spectra, the C=O stretching band of Asn74 in the *p*HtrII­(1-83) was confirmed under the conditions of 0.25, 2.5, and 25 μM concentrations (Figure 6). The results are further evidence of the existence of the PPI in the *p*SRII-*p*HtrII­(1-83) fusion protein in the membrane-embedded region. Concentration-dependent suppression of the positive band at 1641 cm^–1^ and the negative band at 1662 cm^–1^ in the amide I region was observed in *p*SRII-*p*HtrII­(1-83) as well. The reduction was slightly lower than that of the *p*SRII-*p*HtrII­(1-159) especially at 25 μM concentration (compare Figure 4a,b with Figure 6a,b, respectively), which would be interpreted as indicating that the HAMP1 domain contributes partially to the suppression.

**Figure 6:**
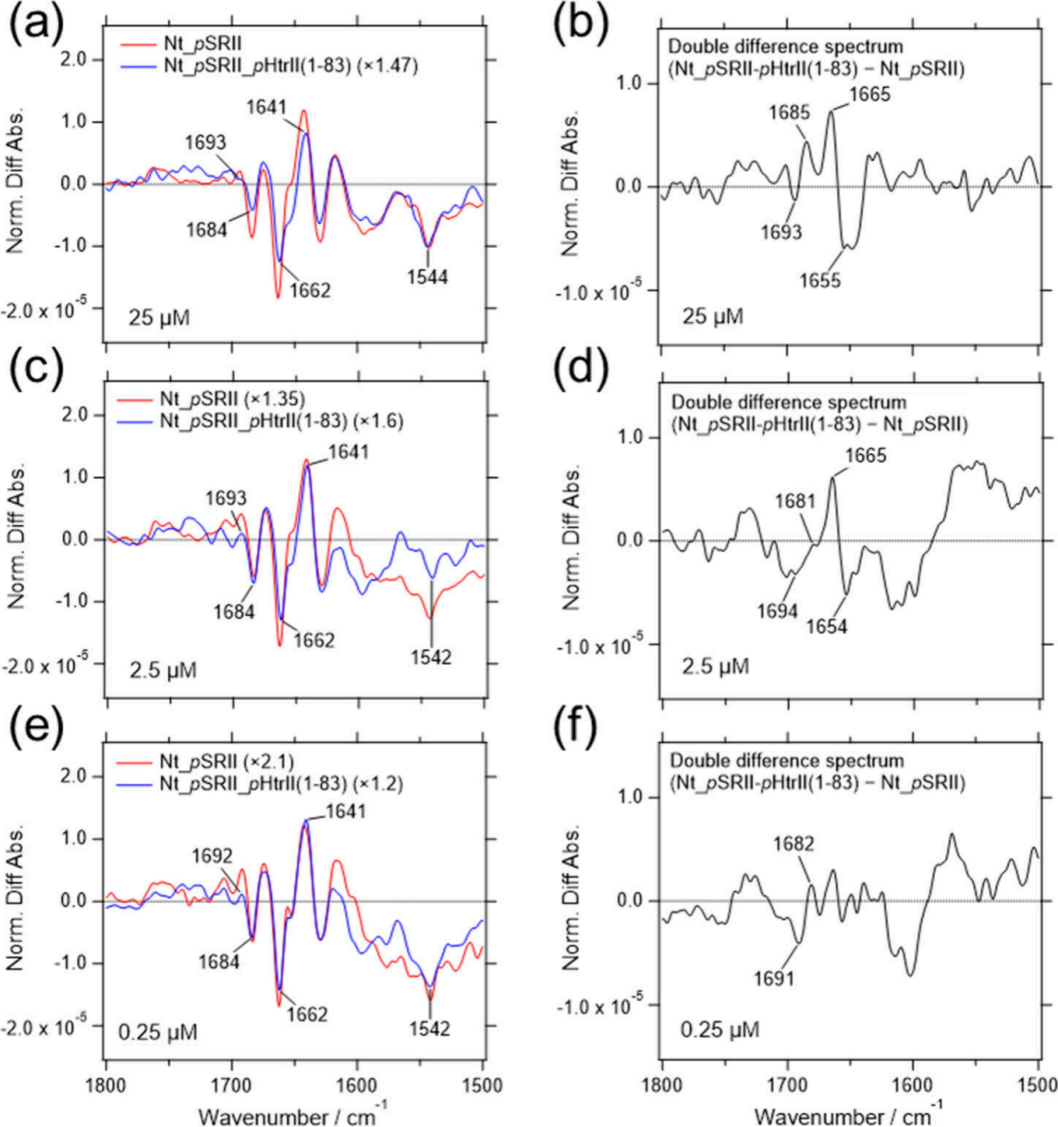
Comparison of the light-induced difference spectra of *p*SRII-*p*HtrII­(1-83) after normalization The light-induced difference spectra (a, c, and e in the left panel) were normalized based on the substantial amplitude of the negative peak from the retinal ethylenic C=C stretching vibration at 1543 cm^–1^. The normalization factors are shown in parentheses after the sample names. The double difference spectra (b, d, and f in the right panel) were calculated by subtracting the difference spectrum of *p*SRII from those of the *p*SRII-*p*HtrII­(1-83). The sample concentrations for adsorption were 25, 2.5, and 0.25 μM as indicated in each panel.

To confirm the validity of the *p*SRII-*p*HtrII fusion constructs, the PPIs between the *p*SRII and truncated *p*HtrII domains were analyzed by flash photolysis spectroscopy (Figure S10). The decay kinetics of the M intermediate of *p*SRII was reported to be delayed in the formation of the complex with a truncated *p*HtrII.^9,11,27^ Flash photolysis data of *p*SRII, *p*SRII-*p*HtrII­(1-83), and *p*SRII-*p*HtrII­(1-159) were recorded at 390 (M intermediate) and 500 nm (the dark state). The sample concentrations were approximately equal to 10 μM. The time constants were obtained by fitting the data with multiple exponential functions (Table S2). The rise kinetics (τ_1_ and τ_2_) of the M intermediate are almost the same among the three samples. However, the decay kinetics (τ_3_ and τ_4_) of the M intermediates were different from each other. The weighted average of the M decay time constants (τ_M_) increased from 0.61 s in *p*SRII to 0.88 s in *p*SRII-*p*HtrII­(1-83) and 0.99 s in *p*SRII-*p*HtrII­(1-159) (Table S2). The value of the *p*SRII was almost the same as the previous experiment (0.60 s), although the value of *p*SRII-*p*HtrII­(1-159) was slightly lower than the previous experiment with the complex of *p*SRII and *p*HtrII­(1-159) prepared separately (1.22 s).^11^ A previous experiment reported that *p*SRII did not bind to *p*HtrII­(1-83) due to the low affinity.^18^ Therefore, the effect of *p*HtrII­(1-83) on the decay kinetics of the M intermediate has not been clarified so far. It is interesting that the decay time constant of *p*SRII-*p*HtrII­(1-83) was between those of the other two. These results support the notion that the PPIs between *p*SRII and the truncated *p*HtrII in the membrane-embedded domains mainly affect the decay kinetics of the M intermediate *p*SRII in the fusion proteins.

## 4 Discussion

Two molecules of *p*SRII and *p*HtrII form a 2:2 complex, and three sets of the 2:2 complex gather to form a trimer of dimers, as reported in previous studies^50,54,55^ (Figure 7a). Furthermore, six sets of the trimers form an O-shaped ring structure even in detergent micelles.^54^ In addition to the formation of the large quaternary structure of the *p*SRII and *p*HtrII complexes, two types of protein conformation were reported in the transmembrane region (U and V shapes) by X-ray crystallography.^36,53^ These experimental aspects would be useful for understanding the SEIRAS experiments in this study.

**Figure 7:**
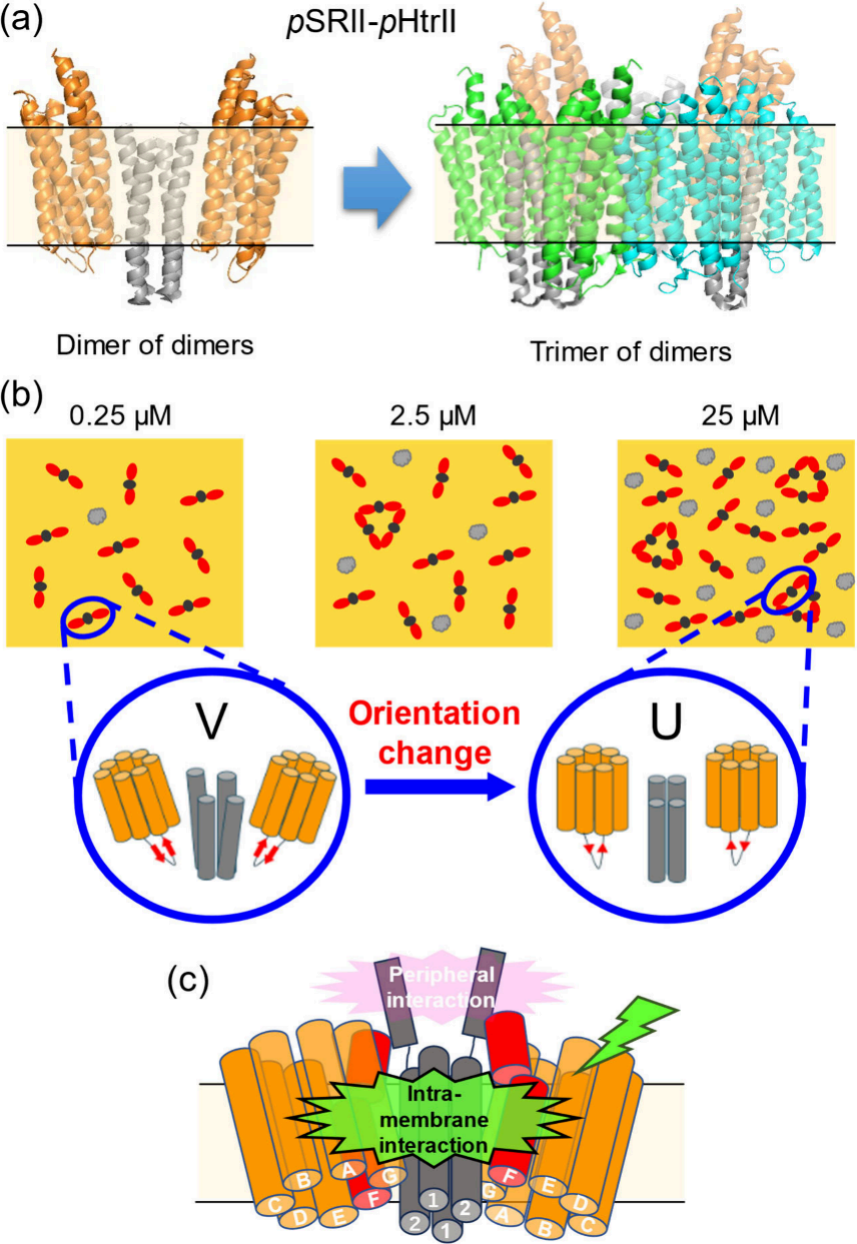
X-ray crystal structure of the *p*SRII/ *p*HtrII complex and a putative model of the trimer of dimers (a) and schematic figures explaining the surface density-dependent conformation change of the *p*SRII and *p*HtrII complex (b) and its photoinduced structural change (c) (a) Three 2:2 complexes of *p*SRII and *p*HtrII (dimer of dimers) gather to form a trimer (trimer of dimers). The ribbon models of *p*SRII and *p*HtrII from PDB entry 1H2S are shown in orange and gray. Additional *p*SRII and *p*HrtII are duplications and are colored green and cyan. (b) Two conformers (V- and U-shaped) were reported by X-ray crystallography, which change the orientations of α helices that make up *p*SRII and *p*HtrII. The formation of the trimer of dimers and the conformation changes may depend on the surface density of *p*SRII and *p*HtrII molecules. Contaminate proteins are depicted by gray objects. (c) SEIRAS demonstrated that the light-induced conformation change of *p*SRII is mainly suppressed by intramembrane interaction with *p*HtrII. The cylinder models of *p*SRII and *p*HtrII in the same color as (a) are shown and the F-helices of *p*SRII are colored in red.

SEIRAS monitored surface density-dependent spectral changes in the amide I region of the *p*SRII-*p*HtrII fusion proteins. *p*SRII-*p*HtrII­(1-159) contains a HAMP1 domain, and *p*SRII-*p*HtrII­(1-83) does not. Therefore, the increases in the amide I band at 1637 cm^–1^, which is characteristic of β sheet structure, cannot be assigned to the change in conformation of the HAMP1 domain. According to the literatures, *p*SRII and *p*HtrII are basically composed of α helical structures.^46,47,49,52,55–57^ Therefore, it was very unlikely that these α-helical structures would convert to β sheet structures. The HAMP1 domain is a flexible region and the atomic structure of the domain was not solved by X-ray crystallography.^36^ However, analysis using nuclear magnetic resonance (NMR) spectroscopy in solution state revealed that the HAMP1 domain is also composed of helical structures.^41^ At the beginning of this study, we speculated that the HAMP1 domain can be converted to β sheet structures in the trimer form. This possibility was completely abolished by the fact that *p*SRII-*p*HtrII­(1-83) increases the amide I band at 1637 cm^–1^ as similarly to *p*SRII-*p*HtrII­(1-159).

It could also be possible that, in the case of the *p*SRII-*p*HtrII fusion protein, partial unfolding may occur due to structural instability within the *p*HtrII region. Moreover, in the SEIRA spectra, the amide I/amide II ratio is not as high as would be predicted based on the orientation selectivity of SEIRAS. However, in the light-induced difference spectra obtained by SEIRA, the C=C stretching vibration of the retinal polyene chain appears relatively small, whereas the amide I changes are pronounced, as compared with the conventional light-induced difference spectra shown in Figure S9. These observations suggest that at least for the photoactive fractions of *p*SRII and the *p*SRII-*p*HtrII fusion protein, the proteins are indeed oriented within the lipid membrane.

To understand the SEIRAS results for the light-induced difference spectra of *p*SRII-*p*HtrII fusion proteins, we propose a working hypothesis that the *p*SRII-*p*HtrII fusion proteins change their quaternary structures in response to increased molecular crowding within the lipid membrane (Figure 7b). In a dispersed condition, most of the α helices in the *p*SRII-*p*HtrII fusion proteins orient almost perpendicularly to the gold surface. However, the absence of neighboring proteins may allow *p*SRII-*p*HtrII in dimers to tilt the helices to some extent (a model in the left circle in Figure 7b). This form would be like the V-shaped form revealed by previous X-ray crystallography.^36^ In proportion to the increase in surface density, the *p*SRII-*p*HtrII fusion proteins form complexes with higher orders such as trimer of dimers.^50,54,55^ In addition, other membrane proteins may act as compressor molecules, increasing the number of tight interactions between *p*SRII-*p*HtrII complexes. This effect was unexpectedly observed in this study, where an unintentionally contaminated β-sheet–rich protein acted as a molecular crowder. In a condensed state, the helices in the *p*SRII-*p*HtrII fusion proteins tend to be tightly packed similar to the U-shaped form reported in the X-ray crystal structure^53^ (a model in the right circle in Figure 7b). The quaternary structural change would be required to reduce the light-induced spectral change in the amide I region.The molecular mechanism of the signal transfer from *p*SRII to *p*HtrII has been extensively studied. Here is the first experimental result obtained by SEIRAS for this topic. In the previous FTIR study, PPI in the membrane domain was detected as a downshift of the C=O stretching vibration of Asn74 in *p*HtrII.^26,27^ Similar spectral changes, probably due to the hydrogen bonding change of Asn74 in *p*HtrII, were observed in both fusion proteins *p*SRII-*p*HtrII­(1-83) and *p*SRII-*p*HtrII­(1-159) independent of surface densities (Figures 4 and 6). Previous FTIR spectroscopy detected suppression of the spectral change in the amide I region (1663/1644 cm^–1^),^26,28^ which is probably a signature of the PPI that the tilt of the F helix of *p*SRII is transferred to the rotation of TM2 in *p*HtrII.^13,21,40^ Spectral attenuation was hampered at low temperature (250 K) and in the complex with *p*HtrII G83C and G83F mutants,^28^ reducing the flexibility of the HAMP1 region. Furthermore, signal transduction in bacterial cells was not observed in the G83C and G83F mutations in *p*HtrII.^14^ Therefore, it was concluded that the HAMP1 domain involves the PPIs related to the tilt of the F-helix in *p*SRII and the rotation of TM2 in *p*HtrII.^28^

In this study, the light-induced difference SEIRAS was applied not only to the *p*SRII-*p*HtrII­(1-159) fusion protein, which is basically similar to the previous FTIR experiment,^27,28^ but also to the *p*SRII-*p*HtrII­(1-83), which is a *p*HtrII truncated before the HAMP1 domain. Unexpectedly, a reduction in the spectral change in the amide I region also occurred in the *p*SRII-*p*HtrII­(1-83) fusion protein. This experimental result strongly suggests that the HAMP1 domain is not important for the PPI regarding the F-helix tilting of *p*SRII and the rotation of TM2 in *p*HtrII. The membrane-embedded region probably plays crucial roles in the PPIs, and the HAMP1 domain plays a minor role (Figure 7c). Our result does not deny that the HAMP1 domain is important in transducing the rotation of TM2 to the intracellular domain of *p*HtrII. The previous FTIR experiments^26,28^ may be interpreted that the low temperature and the G83C and G83F mutations in *p*HtrII inhibited clustering of the *p*SRII/*p*HtrII complex to form higher-order structures such as trimer of dimers and/or the conformation change of the *p*SRII/*p*HtrII complex from V-shaped to U-shaped form, resulting in suppression of the PPI regarding the F-helix tilting of *p*SRII and rotation of TM2 in *p*HtrII. The latter possibility was supported by X-ray crystallography on *p*SRII-*p*HtrII­(1-135: G83F), which exhibited the V-shaped form in the ground state.^53^ Molecular dynamics simulations reported that the U-shaped form in the ground state (closed state) and its conversion to the V-shaped form (open state) upon the formation of the M intermediate represent a key conformational change of the *p*SRII/*p*HtrII complex.^33^ In other words, the V-shaped forms in the ground state of *p*SRII-*p*HtrII­(1-135: G83F) and our *p*SRII-*p*HtrII constructs (1-83 and 1-159) under dispersed conditions do not efficiently transmit the light-induced conformational changes to the *p*HtrII regions.

The changes observed in the amide I region for the *p*SRII-*p*HtrII fusion proteins—showing surface-density dependent attenuation of amide I bands characteristic of α-helices, are, to our knowledge, the first such features reported in SEIRA measurements. We speculate that this phenomenon may be explained by the specific orientation imposed in SEIRAS, where the fusion proteins are aligned through Ni-NTA binding with the extracellular side facing the surface. This orientation is different from that in liposome-based conditions, and such differences may account for the spectral behavior observed here.

## 5 Conclusions

Protein–protein interactions between *p*SRII and *p*HtrII were analyzed by SEIRAS for the first time. The light-induced spectral changes of the *p*SRII-*p*HtrII fusion proteins were highly dependent on their surface density, suggesting that a substantial quaternary structural reorganization—such as the formation of a trimer of dimers, may occur as molecular crowding increases. Furthermore, after normalizing the reduction in spectral intensity caused by dilution with the β-sheet–rich protein acting as a molecular crowder, the light-induced spectral changes of the *p*SRII-*p*HtrII fusion proteins in the amide I region were attenuated under the condensed conditions, regardless of the presence or absence of the HAMP1 domain. These results suggest that suppression of the F-helix tilt in *p*SRII does not require an interaction with the HAMP1 domain. This study provides a new aspect of the molecular mechanism of signal transfer from *p*SRII to *p*HtrII on the basis of the results obtained by SEIRAS applied to the *p*SRII and *p*HtrII fusion proteins under physiological orientation in the membrane.

## Supplementary Material



## Notes

Amino acid sequences of *p*SRII and *p*SRII-*p*HtrII fusion proteins, SEIRA spectra and time courses at the amide I region recorded during adsorption process of *p*SRII and *p*SRII-*p*HtrII fusion proteins, conventional IR spectra of *p*SRII and *p*SRII-*p*HtrII reconstituted into egg-PC liposomes, UV–visible spectra of *p*SRII and *p*SRII-*p*HtrII in detergent solubilized condition, light-induced difference SEIRA spectra of *p*SRII under 500 and 560 nm irradiation, light-induced difference IR spectra of *p*SRII and *p*SRII-*p*HtrII fusion proteins in egg-PC liposomes and their double difference spectra, flash photolysis experiments on *p*SRII and *p*SRII-*p*HtrII fusion proteins, and tables for adsorption process of *p*SRII and *p*SRII-*p*HtrII fusion proteins on the SEIRA substrate and the flash photolysis experiments (PDF)

## Abbreviations

*p*SRII: *pharaonis* sensory rhodopsin 2 (also known as *pharaonis* phoborhodopsin)
*p*HtrII: *pharaonis* halobacterial transducer protein 2
PPI: protein–protein interaction
HAMP: histidine kinase, adenylate cyclases, methyl accepting proteins and phosphatases
FTIR: Fourier transform infrared
SEIRAS: surface-enhanced infrared absorption spectroscopy
Ni-NTA: nickel-nitrilotriacetic acid.
