# Supplementary material for: Surface Density-Dependent Interactions between Photoactivated Sensory Rhodopsin 2 and Its Transducer
Source: ACS Omega. 2026 Feb 2;11(6):10478–91. doi: 10.1021/acsomega.5c12030 (PMC12917780; doi:10.1021/acsomega.5c12030)
Supplement: Supplementary file 1 [file ao5c12030_si_001.pdf]

Supporting information

Surface density-dependent interactions between photoactivated sensory rhodopsin 2 and its transducer

*Tatsuya Sakamoto[1,#], Jingyi Tang,[1,#], Soichiro Kato[1,#], Insyeerah Binti Muhammad Jauhari[1], Tatsuro Nishikino[1,2] and Yuji Furutani[1,2]**

[1]Department of Life Science and Applied Chemistry, Nagoya Institute of Technology, Showa-ku, Nagoya 466-8555, Japan.

[2]OptoBioTechnology Research Center, Nagoya Institute of Technology, Showa-ku, Nagoya 466-8555, Japan.

# These authors contributed equally to this work

* Correspondence author's E-mail: furutani.yuji@nitech.ac.jp

List of the contents in the supporting information

Figure S1: Amino acid sequences of *p*SRII and *p*SRII-*p*HtrII fusion proteins

Figure S2: Infrared absorption spectra in the amide I and II regions recorded by SEIRAS during adsorption of *p*SRII and *p*SRII-*p*HtrII(1-159) with concentrations of 0.25, 2.5, and 25μM

Figure S3: Time courses of increase of the amide I bands at 1654 and 1635 cm^{-1} during adsorption of *p*SRII and *p*SRII-*p*HtrII(1-159) on the Ni-NTA-modified gold surface recorded by SEIRAS

Figure S4: Infrared absorption spectra in the amide I and II regions and their second derivatives of *p*SRII and *p*SRII-*p*HtrII(1-159) reconstituted into egg-PC liposomes recorded by conventional FTIR spectroscopy

Figure S5: UV-vis absorption spectra of *p*SRII, *p*SRII-*p*HtrII(1-83), and *p*SRII-*p*HtrII(1-159) in detergent solubilized conditions

Figure S6: Infrared absorption spectra of *p*SRII and *p*SRII-*p*HtrII(1-159) in the 3800–1450 cm^{-1} region recorded by SEIRAS after binding to the Ni-NTA-modified gold surface

Figure S7: Infrared absorption spectra in the amide I and II regions and C-H stretching vibrations recorded by SEIRAS after reconstitution into egg-PC lipid bilayer

Figure S8: Light-induced difference infrared spectra of *p*SRII recorded by SEIRAS upon irradiation of monochromatic light through interference optical filters of 500 and 560 nm and the double difference spectrum constructed by subtracting the 560-nm spectrum from the 500-nm one

Figure S9: Light-induced difference infrared spectra of *p*SRII and *p*SRII-pHtrII(1-159) reconstituted in egg-PC liposomes recorded by conventional light-induced difference FTIR spectroscopy and their double difference spectrum

Figure S10: Flash photolysis experiments on *p*SRII, *p*SRII-*p*HtrII(1-83), and *p*SRII-*p*HtrII(1-159) in detergent solubilized conditions

Table S1: Time constants for the rise kinetics recorded at 1654 (or 1651) and 1635 cm^{-1} for *p*SRII and *p*SRII-*p*HtrII(1-159) adsorption with concentrations of 0.25, 2.5, and 25 μM

Table S2: Time constants obtained from the global exponential fitting on the flash photolysis data recorded at 390 and 500 nm for *p*SRII, *p*SRII-*p*HtrII(1-83), and *p*SRII-*p*HtrII(1-159)

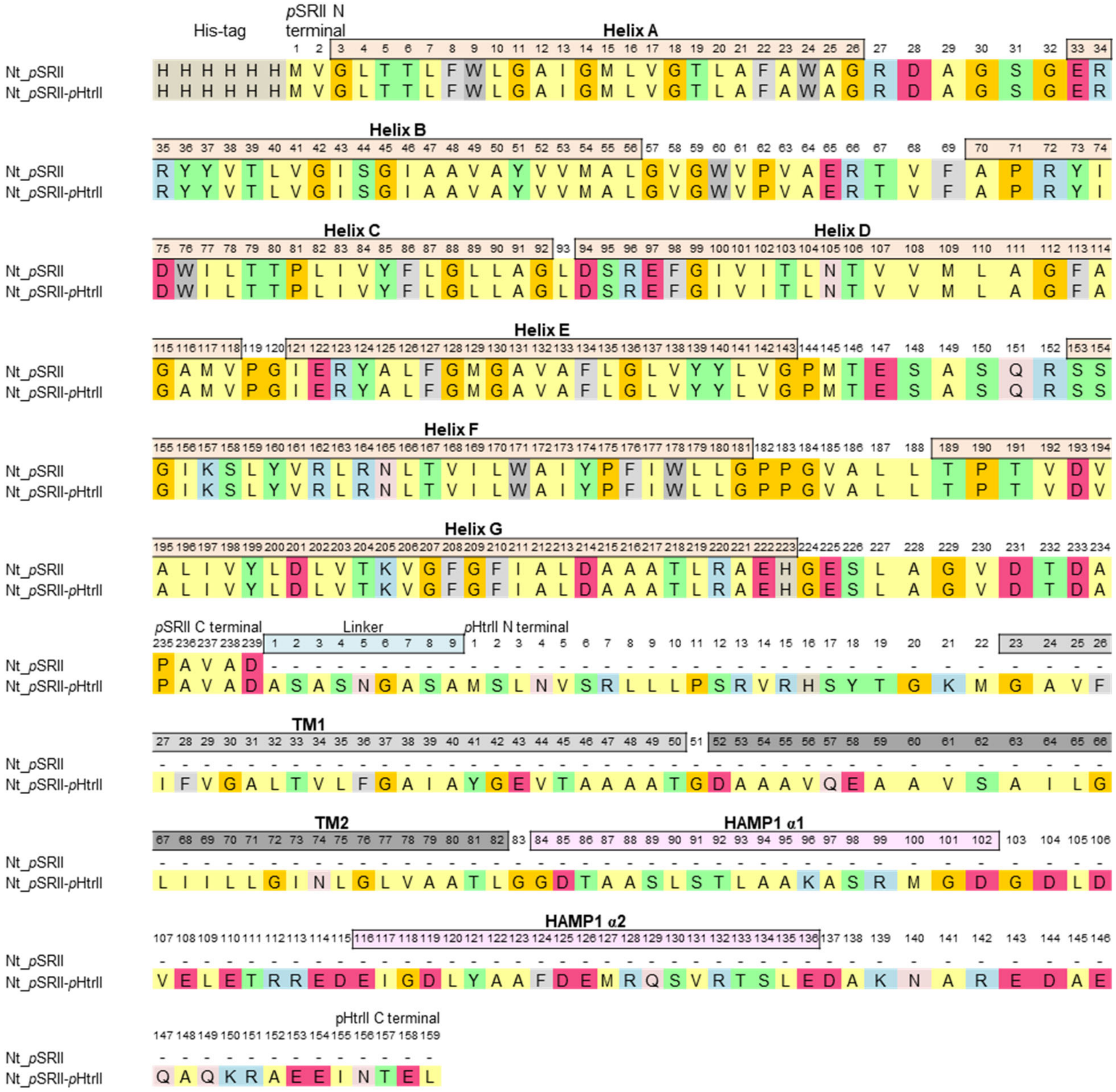

Figure S1. Amino acid sequences of *p*SRII and *p*SRII-*p*HtrII fusion proteins Six histidine residues (His-tag) are introduced at the N-terminus for purification and binding to the Ni-NTA linkers modified on the gold surface for SEIRA experiments. *p*HtrII is connected via a linker (ASASNGASA) with the C-terminus of *p*SRII. *p*SRII-*p*HtrII(1-159) contains the HAMP1 domain, while *p*SRII-*p*HtrII(1-83) does not.

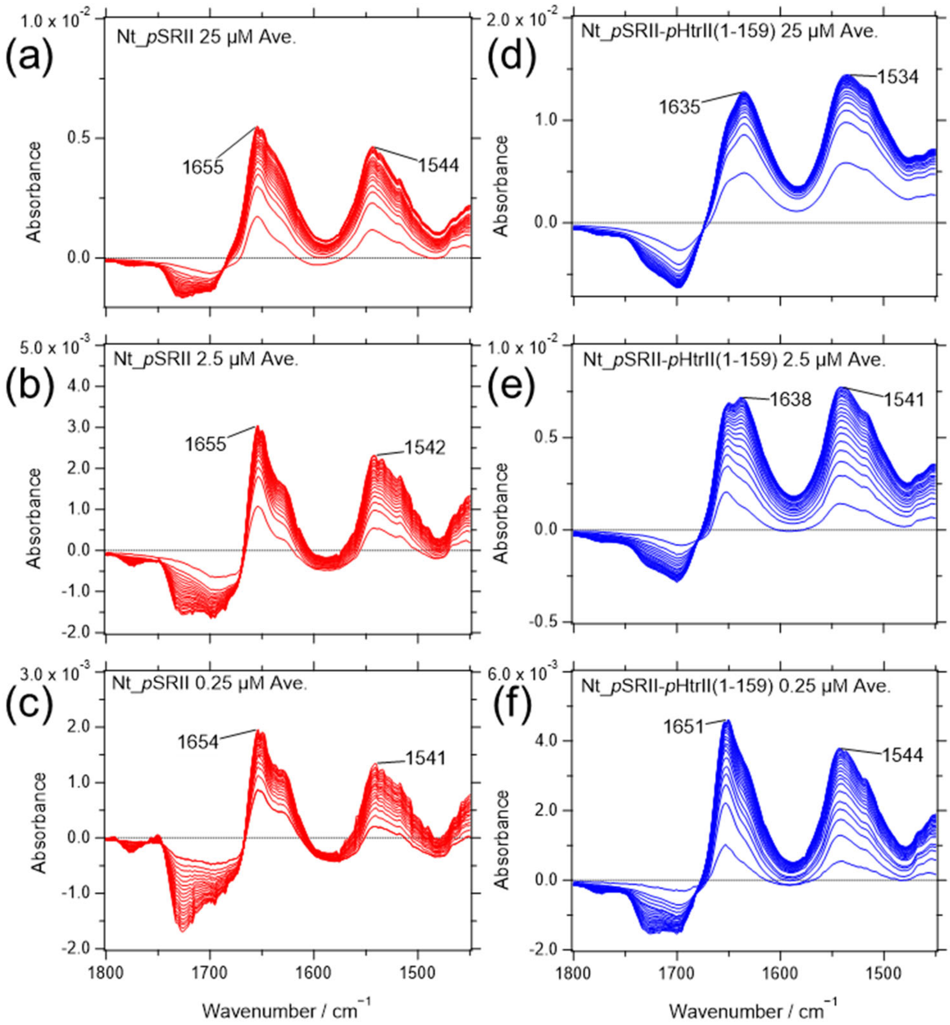

Figure S2. Infrared absorption spectra in the amide I and II regions recorded by SEIRAS during the adsorption of *p*SRII (left panel) and *p*SRII-*p*HtrII(1-159) (right panel) with concentrations of 0.25 (a, d) , 2.5 (b, e) and 25 (c, f) µM The spectra were recorded with a 5-min interval.

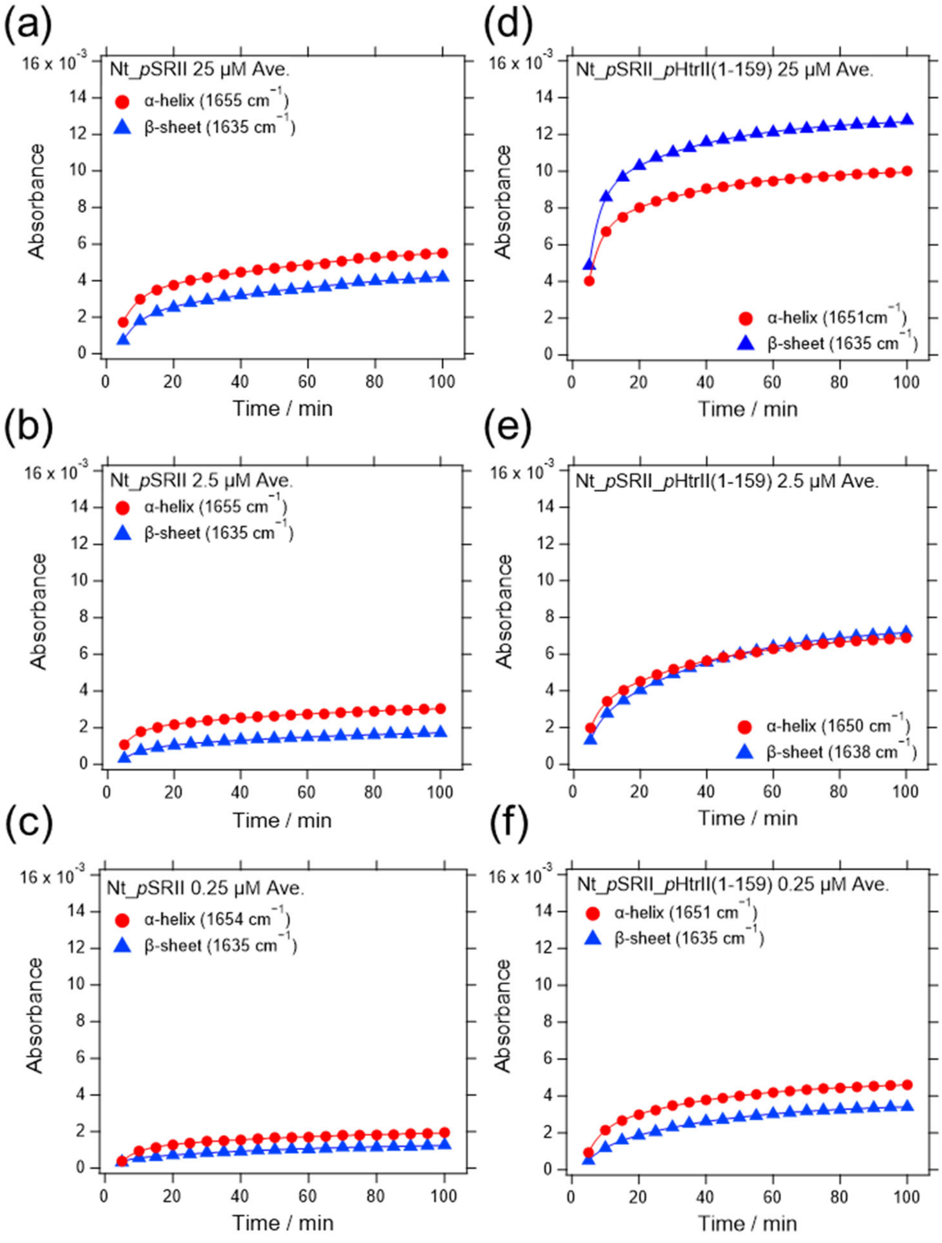

Figure S3. Time courses of increase of the amide I bands at 1654 and 1635 cm^{-1} during adsorption of *p*SRII (left panel) and *p*SRII-*p*HtrII(1-159) (right panel) on the Ni-NTA-modified gold surface recorded by SEIRAS The sample concentrations of *p*SRII (a, b, c) and *p*SRII-*p*HtrII(1-159) (d, e, f) were 0.25, 2.5, and 25 μM, respectively. The traces are the averages of three experiments under each condition. The time constants for the rise kinetics recorded at 1654 (or 1651) and 1635 cm^{-1} are listed in Table S1.

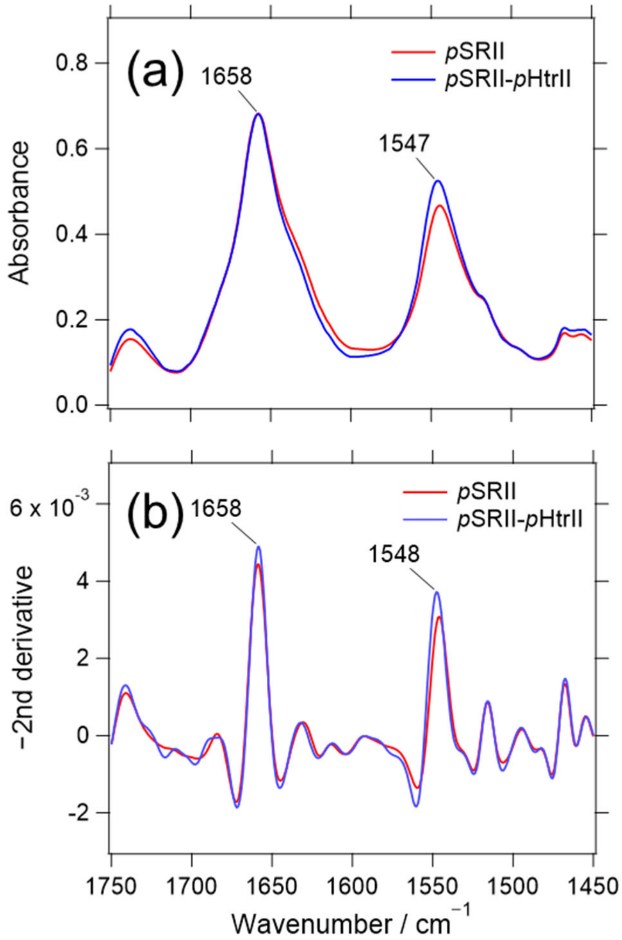

Figure S4. Infrared absorption spectra (top panel) in the amide I and II regions and their second derivatives (bottom panel) of *p*SRII and *p*SRII-*p*HtrII(1-159) reconstituted into egg-PC liposomes recorded by conventional FTIR spectroscopy Two peaks at 1658 and 1547 (or 1548) cm^{-1} are tagged as amide I and II bands, respectively.

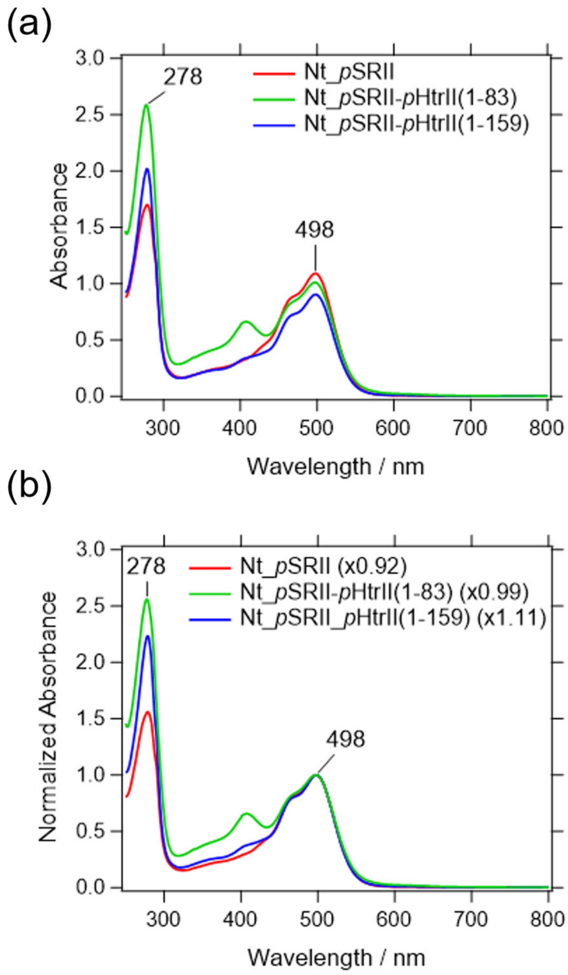

Figure S5. UV-vis absorption spectra of *p*SRII, *p*SRII-*p*HtrII(1-83), and *p*SRII-*p*HtrII(1-159) in the detergent solubilized conditions (a). The normalized spectra at 498 nm are shown in (b). The ratios between absorbance at 278 and 498 nm are 1.56 (*p*SRII), 2.56 (*p*SRII-*p*HtrII(1-83)), and 2.23 (*p*SRII-*p*HtrII(1-159)).

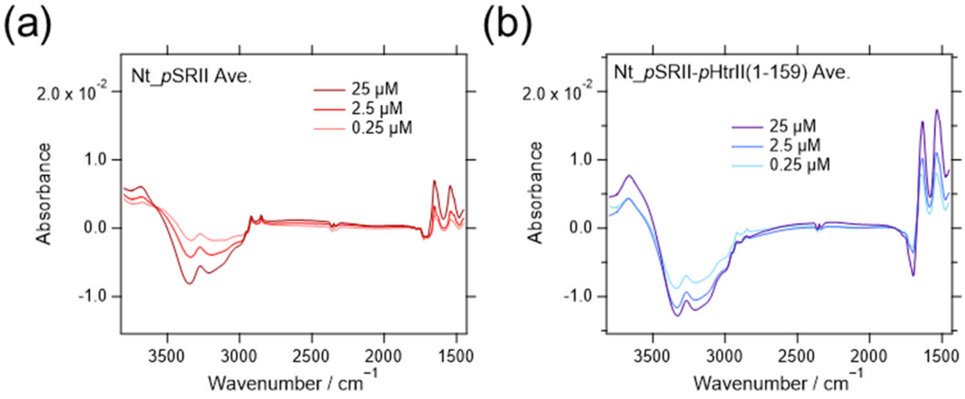

Figure S6. Infrared absorption spectra of *p*SRII and *p*SRII-*p*HtrII(1-159) in the 3800–1450 cm^{-1} region recorded by SEIRAS after binding to the Ni-NTA-modified gold surface. These spectra are reproduced from Figure 2 (a, b) with an extended frequency range. The sample concentrations of *p*SRII (a) and *p*SRII-*p*HtrII(1-159) (b) were 0.25, 2.5, and 25 μM, respectively.

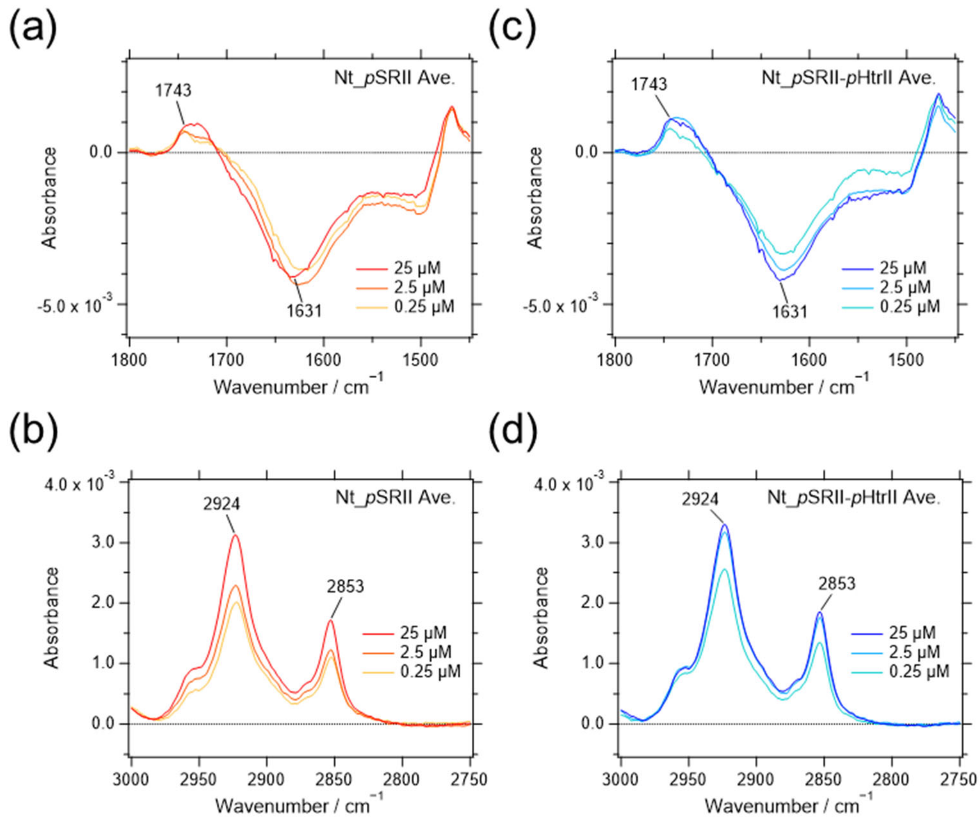

Figure S7. Infrared absorption spectra in the amide I and II regions (top panel) and C-H stretching vibrations (bottom panel) recorded by SEIRAS after reconstitution into egg-PC lipid bilayer The sample concentrations of *p*SRII (a, b) and *p*SRII-*p*HtrII(1-159) (c, d) were 0.25, 2.5, and 25 µM, respectively.

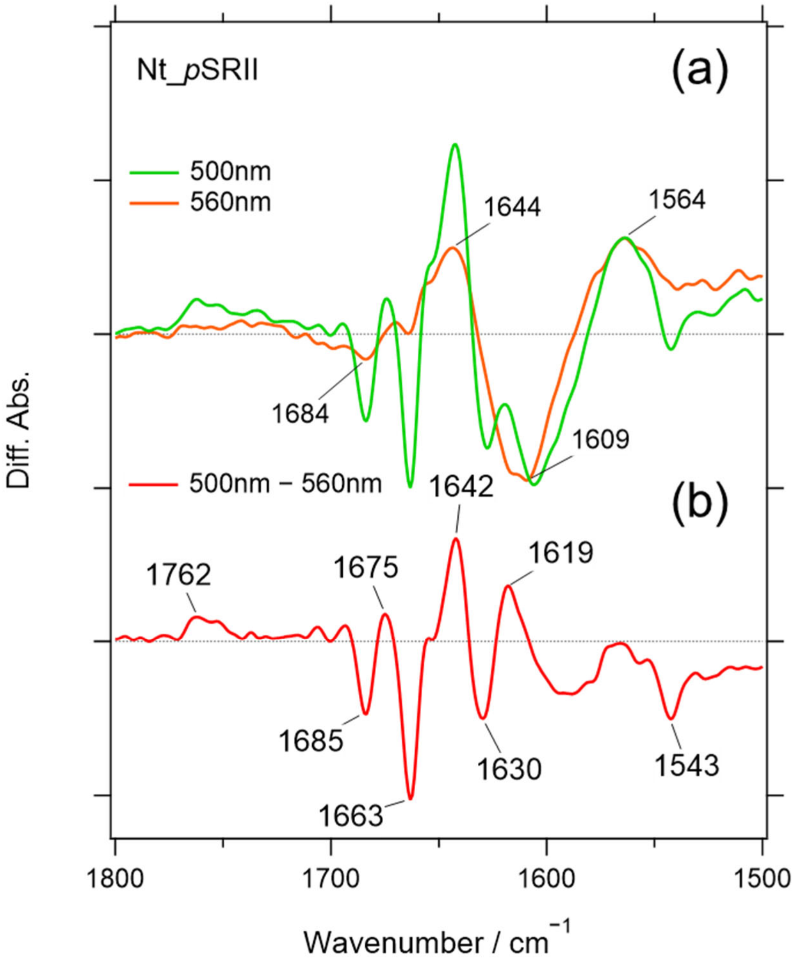

Figure S8. Light-induced difference infrared spectra of *p*SRII (a) recorded by SEIRAS upon irradiation of monochromatic light through interference optical filters of 500 and 560 nm and the double difference spectrum (b) constructed by subtracting the 560-nm spectrum from the 500-nm one. The bands characteristic to *p*SRII are tagged with their wavenumbers.

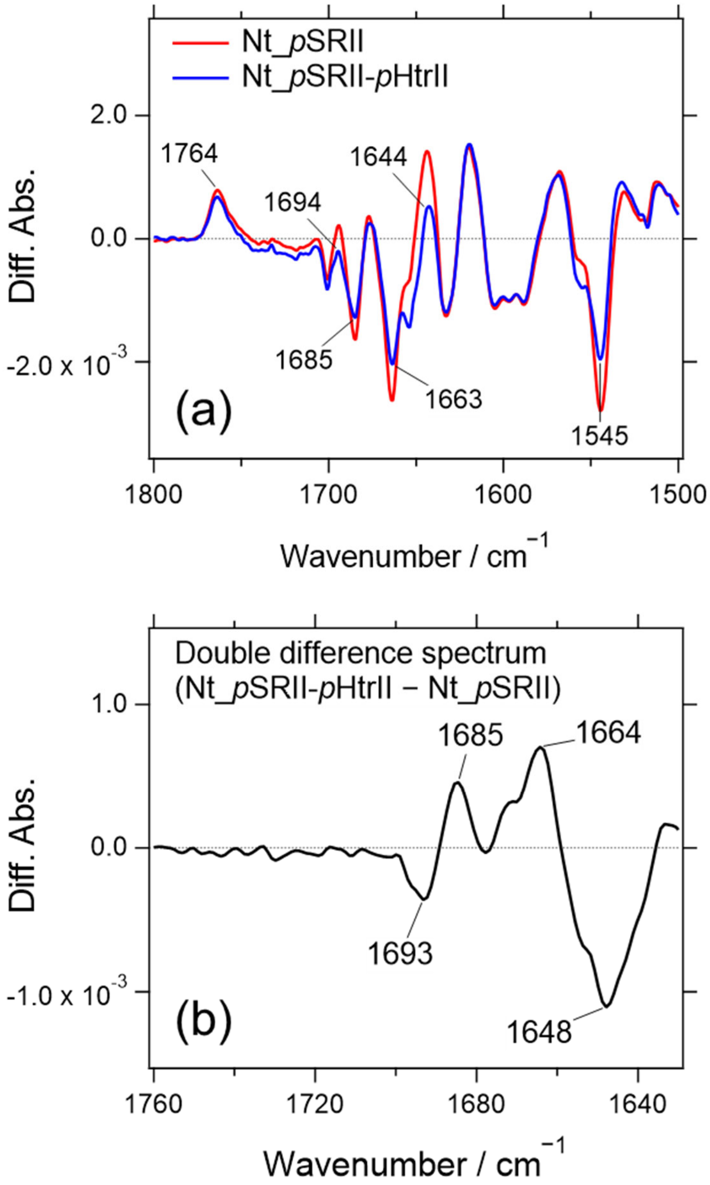

Figure S9. Light-induced difference infrared spectra of *p*SRII and *p*SRII-*p*HtrII(1-159) reconstituted in egg-PC liposomes recorded by conventional light-induced difference FTIR spectroscopy (a) and their double difference spectrum (b)

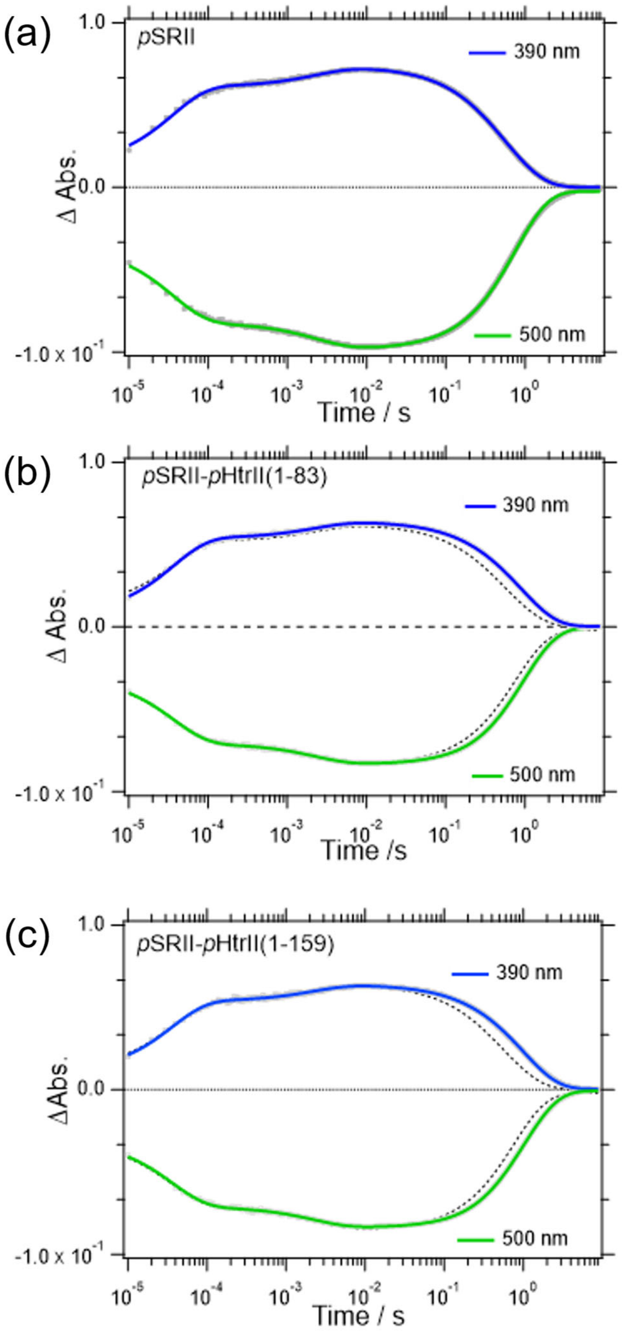

Figure S10. Flash photolysis experiments on *p*SRII (a), *p*SRII-*p*HtrII(1-83) (b), and *p*SRII-*p*HtrII(1-159) (c) under detergent-solubilized conditions. Absorption changes at 390 and 500 nm were recorded (gray dots) and fitted by four exponential functions (blue; 390 nm, green; 500 nm). The absorbances at the λ_{max} are 0.474 for *p*SRII, 0.401 for *p*SRII-*p*HtrII(1-83), and 0.416 for *p*SRII-*p*HtrII(1-159). Protein concentrations were approximately 10 μM for all samples. The dashed lines in (b) and (c) are duplications of the fitted curves for *p*SRII with normalization (0.85 for (b) and 0.87 for (c)). The time constants are listed in Table S2.

Table S1. The time constants for the rise kinetics recorded at 1654 (or 1651) and 1635 cm^{-1} for *p*SRII and *p*SRII-*p*HtrII(1-159) adsorption with concentrations of 0.25, 2.5, and 25 μM The experimental data are shown in Figure S2 and S3. Two exponential functions were used to fit each data set. Weighted averages of τ_1 and τ_2 were calculated for the overall rise time constants for the adsorption reactions. The errors were estimated from the global exponential fitting.

		τ_1 (s)	τ_2 (s)	τ_{rise} (s)
~1654 cm^{-1}				
*p*SRII	0.25	5.1 ± 0.8	59 ± 14	25
	2.5	4.3 ± 0.5	64 ± 9	26
	25	4.8 ± 0.5	81 ± 9	37
*p*SRII-*p*HtrII(1-159)	0.25	4.2 ± 0.3	38 ± 2	18
	2.5	3.7 ± 0.3	37 ± 1	20
	25	3.3 ± 0.3	33 ± 2	10
~1635 cm^{-1}				
*p*SRII	0.25	2.3 ± 1.7	52 ± 7	22
	2.5	5.5 ± 0.8	70 ± 13	36
	25	5.3 ± 0.6	79 ± 14	39
*p*SRII-*p*HtrII(1-159)	0.25	4.0 ± 0.9	36 ± 2	25
	2.5	3.0 ± 0.3	36 ± 0.6	21
	25	3.3 ± 0.2	32 ± 2	9

Table S2. Time constants obtained from the global exponential fitting on the flash photolysis data recorded at 390 and 500 nm for *p*SRII, *p*SRII-*p*HtrII(1-83), and *p*SRII-*p*HtrII(1-159) The experimental data are shown in Figure S7. Four exponential functions were used for the global fitting of each data set. The weighted averages of τ_3 and τ_4 were calculated for the overall decay time constants for M intermediate (τ_M). The errors were estimated from the global exponential fitting.

	τ_1 (μs)	τ_2 (ms)	τ_3 (s)	τ_4 (s)	τ_M (s)
*p*SRII	35.6 ± 0.5	2.44 ± 0.03	0.28 ± 0.01	0.701 ± 0.004	0.61
*p*SRII-*p*HtrII(1-83)	36.5 ± 0.4	2.34 ± 0.03	0.19 ± 0.01	0.923 ± 0.003	0.88
*p*SRII-*p*HtrII(1-159)	36.2 ± 0.4	2.32 ± 0.03	0.15 ± 0.01	1.019 ± 0.003	0.99

Materials and Methods used for the data shown in the supporting information.

Conventional light-induced difference FTIR experiments

Light-induced difference FTIR spectroscopy was performed similarly to previous experiments (K. Kamada et al. *Biochemistry* 45(15):4859-4866, 2006). The *p*SRII and *p*SRII-*p*HtrII(1-159) samples reconstituted into egg-PC liposomes (protein-lipid molar ratio is 1:50) were washed three times with 2 mM NaH_2PO_4 (pH 7.0). The pellet was resuspended in the same buffer and adjusted to a concentration of 2.5 mg mL^{-1}. A 60 μL aliquot was placed on a BaF_2 window and dried using an aspirator. The samples were hydrated with H_2O prior to analysis. The hydrated sample was mounted on a sample holder and placed in a cryostat (Optistat DN2, Oxford Instruments) attached to an FTIR spectrometer (Cary 670, Agilent Technologies Japan, Ltd.). The sample temperature was kept at 293 K using liquid nitrogen, with a temperature regulation at ±0.1 K precision. The M intermediate was accumulated by continuous illumination with >480 nm light (Y-50, Toshiba interference filter) from a 300 W Xenon lamp (Max-303, Asahi Spectra) during the measurement for the light condition. The interferograms of the sample were collected in the dark and during illumination with 128 scans. Infrared spectra were Fourier transformed with a spectral resolution of 2 cm^{-1}. Ten spectra were averaged for dark and light conditions. The light-induced difference spectra were calculated from the averaged spectra.

Flash photolysis experiments

*p*SRII, *p*SRII-*p*HtrII(1-83), and *p*SRII-*p*HtrII(1-159) samples were resuspended in a buffer containing 100 mM NaH_2PO_4 (pH 7.0), 150 mM NaCl, and 0.1%(w/v) DDM. A 0.6 mL aliquot of each sample was placed in a cuvette (1 cm pathlength) held in a temperature controlled holder (Flash300, Quantum Northwest, USA). The absorbance at the λ_{max} of *p*SRII, *p*SRII-*p*HtrII(1-83), and *p*SRII-*p*HtrII(1-159) were 0.474 (499 nm), 0.401 (500 nm), and 0.416 (499 nm), respectively. The sample temperature was maintained at 20°C using an embedded Peltier device and a circulating water jacket connected to a thermostat bath (Alpha RA8, Lauda). Each sample was illuminated with a tunable laser (490 nm) at energy of 1.0 mJ per pulse with an optical parametric oscillator (LT-2214, LOTIS TII) which was connected with a nanosecond-pulsed laser from second-harmonic generation of an Nd:YAG laser (LS-2134UT-10, LOTIS TII). The repetition rate was adjusted to 12 s, which is sufficient to complete the photocycle of each sample, preventing unintended photoexcitation of transient intermediates. The absorption changes following laser excitation were probed using monochromatic light at 390 or 500 nm, generated by an Xe arc lamp (L9289-01, Hamamatsu Photonics, Japan). Probe light detection was performed using a photomultiplier tube (R10699, Hamamatsu Photonics) connected to a digital storage oscilloscope (MSO22 2-BW-500, Tektronix, Japan) with a resistance of 5 kΩ. Twenty transient data were averaged for each measurement. Time-resolved transient absorption data were analyzed using Igor Pro (version 9) and the Global Fit package, applying multiple exponential functions to determine decay time constants and preexponential factors.
